# Hormonal and transcriptomic regulation of drought adaptation in barley roots and leaves

**DOI:** 10.1038/s41598-025-01590-2

**Published:** 2025-05-11

**Authors:** Anna Collin, Magdalena Pacwa-Plociniczak, Tomasz Plociniczak, Ôndrej Novak, Marek Marzec, Wenbin Guo, Craig G. Simpson, Agata Daszkowska-Golec

**Affiliations:** 1 Institute of Biology, Biotechnology and Environmental Protection, Faculty of Natural Sciences, University of Silesia in Katowice, Jagiellońska 28, Katowice, 40-032 Poland; 2 Laboratory of Growth Regulators, Faculty of Science, Palacký University & Institute of Experimental Botany, The Czech Academy of Sciences, Olomouc, Czech Republic; 3 Information and Computational Sciences, James Hutton Institute, Dundee, DD2 5DA Scotland, UK; 4 Cell and Molecular Sciences, James Hutton Institute, Dundee, DD2 5DA Scotland, UK

**Keywords:** Alternative splicing (AS), Barley, Drought, Hormones, Metatranscriptomics, Transcriptomics, RNA splicing, Plant sciences, Plant hormones

## Abstract

Drought poses a significant threat to global crop productivity and food security. In this study, we aimed to elucidate the impact of drought on transcriptional regulation and alternative splicing in barley (*Hordeum vulgare*), and to determine whether these transcriptomic alterations correlate with changes in hormonal profiles. We hypothesized that drought stress induces extensive reprogramming of gene expression, including alternative splicing events, and that these molecular responses are accompanied by tissue-specific shifts in hormone levels, ultimately underpinning adaptive responses in both leaves and roots. To test this, we performed RNA-seq and comprehensive hormone profiling on leaves and roots sampled at 25 days after planting under both optimal and drought conditions. Our analysis identified over 6,655 differentially expressed genes, with a substantial subset exhibiting differential alternative splicing. In leaves, drought primarily downregulated photosynthesis-related genes while upregulating pathways involved in water stress and abscisic acid (ABA) signaling. In contrast, roots displayed broader metabolic adjustments and significant isoform switching. Hormone analysis revealed marked ABA accumulation, particularly in roots, alongside organ-specific modulation of jasmonates and auxins. A limited assessment of the rhizosphere microbial community revealed low transcript abundance, underscoring the primacy of intrinsic plant responses. Collectively, these findings provide valuable insights into the multilayered adaptive strategies of barley under drought stress.

**Supplementary Information:**

The online version contains supplementary material available at 10.1038/s41598-025-01590-2.

## Introduction

Drought stress is a major abiotic factor that negatively affects agricultural productivity^1,2^. Plants have evolved complex adaptive strategies to mitigate and respond to environmental challenges^3,4^. Among these strategies, plant hormones play a crucial role in modulating the responses to drought stress. Abscisic acid (ABA) is central to the drought response, promoting stomatal closure and regulating gene expression to enhance water use efficiency^5,6^. Additionally, drought stress influences the levels of other hormones, such as ethylene, gibberellins, cytokinins, and auxins, which interact to fine-tune plant growth and stress responses^7,8^.

Alternative splicing (AS) is another crucial adaptive mechanism of plants under abiotic stress^9–11^. AS allows plants to produce multiple transcript variants from a single gene, potentially expanding their response to environmental changes^10,12^. Drought stress induces many AS events in plants, indicating that AS is a crucial mechanism for regulating gene expression in response to stress^13,14^. Isoform switching is one type of AS regulation in response to stress. In this scenario, stress activates the expression of some isoforms, whereas the production of others is terminated^15,16^. In Arabidopsis, more than 450 isoform switching events have been detected in response to 15 days of drought^16^. However, there is still a lack of comprehensive studies on crops, such as barley (*Hordeum vulgare*), regarding the simultaneous analysis of the transcriptional and splicing levels of regulation in response to abiotic stresses. Taking advantage of the recently published Reference Transcriptome Dataset BaRTv2.18 for barley^17^ it is possible to track changes at the AS level in barley.

Plant adaptation to drought stress involves diverse physiological and molecular mechanisms, one of which includes interactions with soil microbial communities. Although not the sole factor, the rhizosphere—the soil zone influenced by root exudates—can modulate plant responses, as correlations have been observed between microbial drought response mechanisms and plant adaptation^18^. Moreover, certain microbes produce growth-promoting compounds, such as auxins and 1-aminocyclopropane-1-carboxylic acid (ACC) deaminase^19^ and inoculation with plant growth-promoting rhizobacteria (PGRP) has been shown to enhance crop resilience to drought stress^20–22^.

Barley ranks as the fourth major agronomic crop and plays a vital role in global agricultural systems, making it an essential model for studying plant drought responses^23–25^. While the physiological adjustments of barley to drought have been well characterized, emerging research indicates that drought-induced hormonal changes—such as increased abscisic acid (ABA) levels that promote auxin signaling in roots and support the development of drought-adaptive root structures^26^ - are central to its stress response. In addition, integrating plant and microbial transcriptomic data reveals that drought triggers adaptive processes in both the plant and its associated microbiota^27,28^, offering broader insights into the coordinated strategies underpinning plant resilience under water deficit conditions^18,29,30^.

Here, we wanted to explore what is barley response to water deficit above and below ground. Thus, we integrated transcriptomic and hormonomic analyses to elucidate drought-induced alterations in barley, focusing primarily on roots and leaves while complementing these data with an assessment of the rhizosphere microbiome. We investigated transcriptional reprogramming, alternative splicing, and hormonal dynamics to reveal the mechanisms underlying barley adaptation to water deficit. This integrated approach enhances our understanding of plant drought responses and underscores the value of multi-level analyses for developing climate-resilient crops.

## Results

### Drought elicits extensive transcriptional and alternative splicing reprogramming in barley

To capture both gene-level expression changes and transcript-level regulatory events, we analyzed differentially expressed (DE) genes and differentially expressed transcripts. While our downstream analyses focused on DE genes for functional interpretation and validation, the inclusion of DE transcripts allowed us to detect alternative splicing and isoform switching, providing a more detailed view of the transcriptomic response.

We performed RNA-seq on the leaves and roots of barley. Plants growing under optimal water conditions and under drought were analyzed and sampled at 25 DAP (days after planting).

**Fig. 1:**
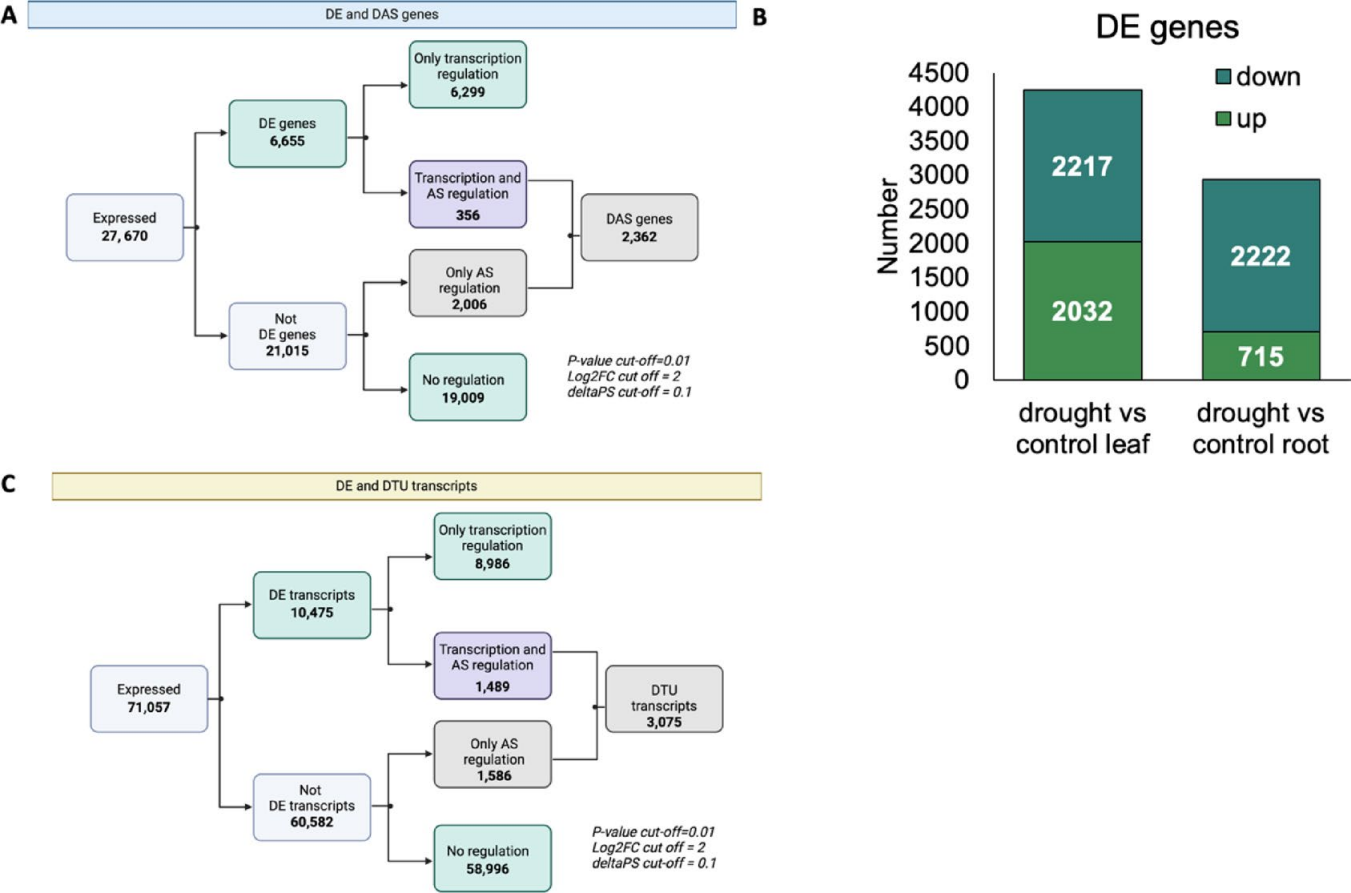
Comparative analysis of drought-induced Differential Expression of Genes (DEGs), Transcripts (DETs), and Alternative Splicing Events (DAS) in barley roots and leaves. (**A**) Differential Expression Analysis at the gene-level; (**B**) Differential Expression Analysis at the transcript-level; (**C**) Union set DE transcripts vs. DTU (Differential Transcript Usage) transcripts.

Over 320 million paired-end reads were generated for each treatment – including all biological replicates. Data were analyzed at the gene and individual transcript levels to identify genes with significant differential expression (DE) and differential alternative splicing (DAS) between drought and control in the roots and leaves. We identified 6655 DE genes (Supplementary Table S1) and 2362 DAS genes (Supplementary Table S2; Fig. 1A). The overlap between DE and DAS genes was 356 genes being both significantly regulated by transcription (DE) and alternative splicing (DAS) in response to drought stress (DE and DAS) (Fig. 1A).

We first looked closer to DE genes and discovered that 47% (2032) of genes were upregulated, whereas 53% (2217) were downregulated in leaves, while in roots, 24% (715) were upregulated, and 76% (2222) were downregulated (Fig. 1B). Of the 6655 genes that exhibited significant differential expression, 35% (2362) were differentially alternatively spliced (DAS). We performed an analysis at the transcript level to identify the individual transcripts responsible for the genes identified as DAS. We identified 10 475 transcripts that showed significant drought-responsive changes (Fig. 1C). Among them, 3075 transcripts showed changes in AS, 1489 were both regulated at transcription and by alternative splicing, and 1586 showed splicing regulation (DTU-only) (Fig. 1C; Supplementary Table S3). Notably, 76% of these transcripts were protein-coding and 21% contained premature termination codons (PTC).

**Fig. 2:**
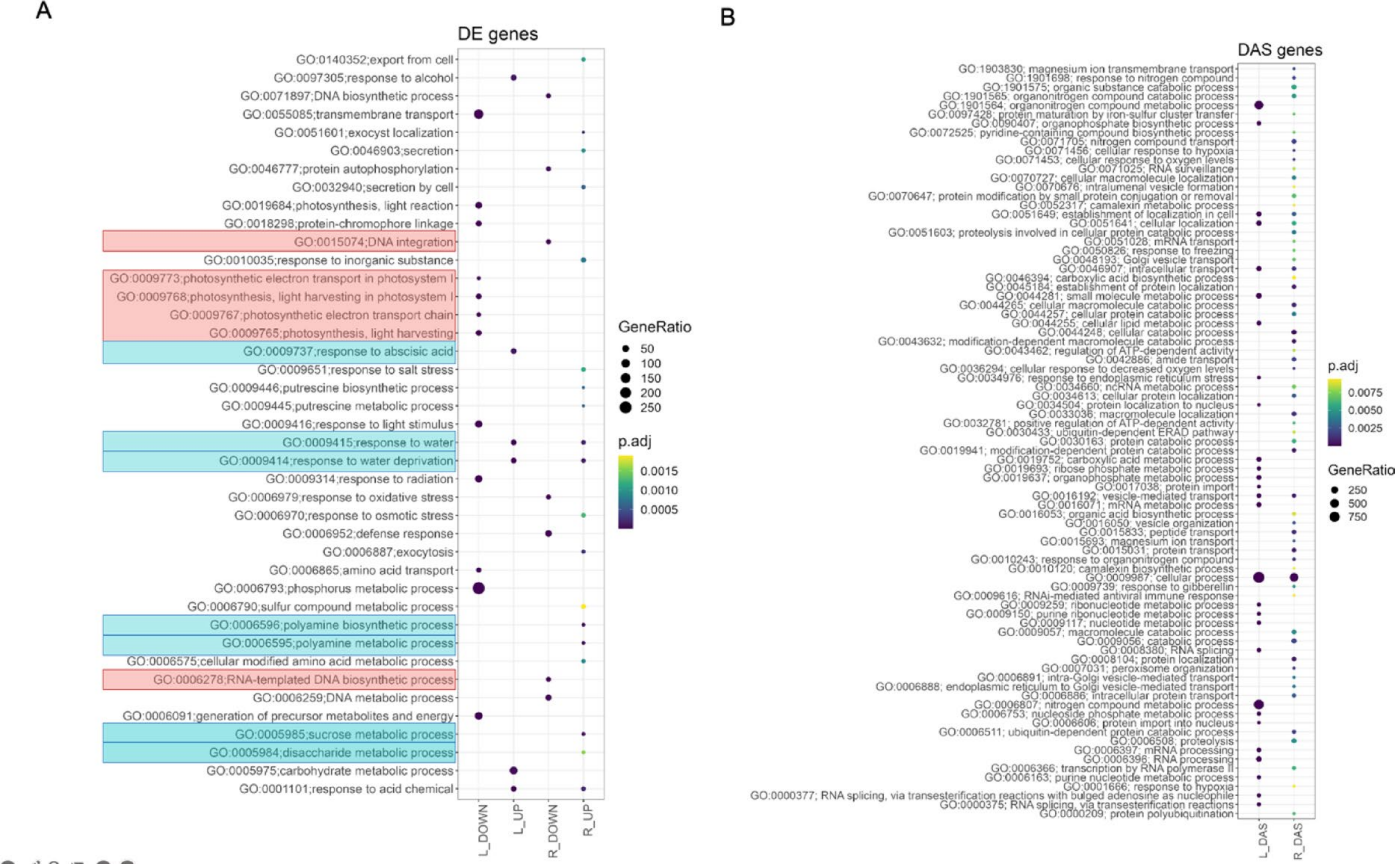
GO enrichment analysis of Differentially Expressed Genes (DEGs) (**A**) and Differentially Alternatively Spliced (DAS) genes (**B**) in response to drought. Biological Processes (BP) are displayed only. L_DOWN/L_UP and R_DOWN/R_UP mean genes down- and upregulated respectively in leaf and in root, L_DAS and R_DAS mean genes differential alternatively spliced (DAS) respectively in leaf and in root. In the red boxes are GO BP tags representing downregulated DEGs, while blue boxes represent GO BP tags represent upregulated DEGs. GeneRatio represents the number of input genes annotated to a given GO term in a reference transcriptome.

GO enrichment analysis of 2006 DAS-only genes (Fig. 1A) showed the importance of splicing as an additional layer of regulation but also allowed us to distinguish between root and leaf responses to drought. To elucidate the molecular mechanisms of drought response in plants, we conducted a thorough Gene Ontology (GO) enrichment analysis of differentially expressed genes (DEGs) (Fig. 2A) and differentially alternatively spliced genes (DAS) (Fig. 2B) in both leaves and roots. Our data revealed organ-specific enrichment patterns, highlighting the unique strategies employed by each organ in response to drought.

### Distinct function of DEGs and DAS in leaf and root under drought

In leaves, downregulated DEGs were predominantly associated with processes related to photosynthesis (GO:0019684), notably light reaction and light harvesting (GO:0009765). The upregulated DEGs were enriched in categories associated with response to water (GO:0009415), water deprivation (GO:0009414), and abscisic acid (GO:0009737) (Fig. 2A; Supplementary Table S1).

Roots exhibit multifaceted responses to drought. Downregulated DEGs were significantly enriched in categories related to DNA integration (GO:0015074) and RNA-templated DNA biosynthesis (GO:0006278). Conversely, upregulated genes were enriched in metabolic processes, particularly those linked to polyamine metabolism (GO:0006595), biosynthetic processes (GO:0006596) and sucrose metabolic process (GO:0005985) (Fig. 2A; Supplementary Table S1).

We also explored the differential alternative splicing (DAS) landscape in both leaves and roots under drought (Fig. 2B). Genes that exhibited differential splicing in leaves were predominantly associated with metabolic processes. Notably, organophosphate metabolic process (GO:0019637) and small-molecule metabolic process (GO:0044281) emerged as the primary categories. Additionally, mRNA processing (GO:0006397) was highlighted. In contrast, roots showed a broader spectrum of enriched GO terms. Response to gibberellin (GO:0009739) and peroxisome organization (GO:0007031) were among the top categories (Fig. 2B; Supplementary Table S2). This suggests splicing engagement in many pathways, from hormonal signaling to cellular reorganization and metabolic tweaks. In the leaf, the primary modulated processes are basic metabolic processes, including mRNA processing, whereas in the root, a broader range of molecular adjustments was observed.

### Drought-responsive expression and AS of transcription factors

By analyzing both differentially expressed (DE) and differentially alternatively spliced (DAS) genes, we identified key transcription factor (TF) families in barley leaf and root that are involved in plant response to drought (Supplementary Table S4).

**Fig. 3:**
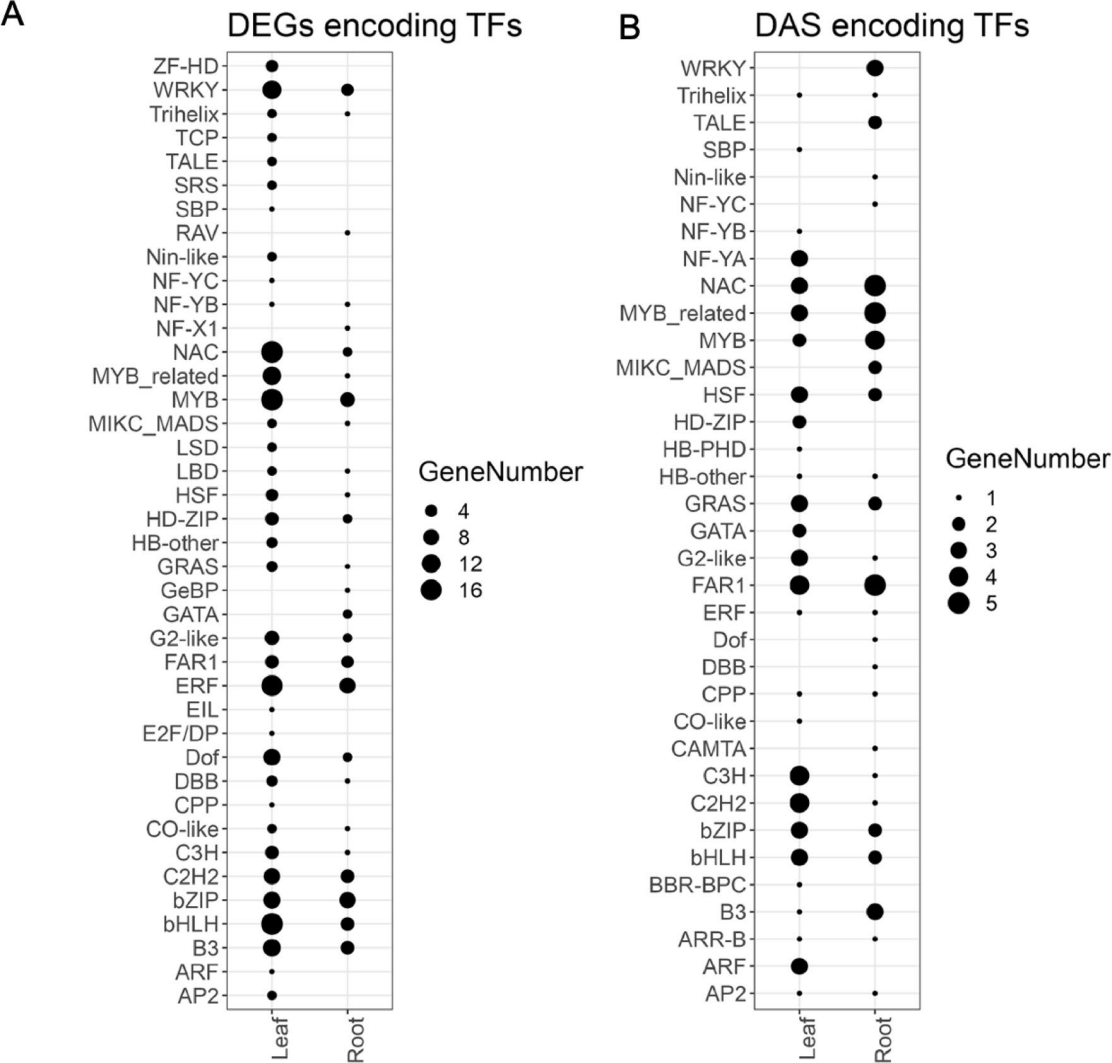
Transcription factors (TFs) encoded by Differentially Expressed Genes (DEGs) (**A**) and Differentially Alternatively Spliced (DAS) (**B**) genes in leaves and roots under drought.

In leaves, the transcriptional landscape under drought illuminated the pivotal role of the bHLH (17 genes), MYB (17), ERF (16), NAC (17), and WRKY (12) TF families (Fig. 3A). The upregulated *HECATE1* (BaRT2v18chr5HG251050) gene, which belongs the bHLH family, showed an 8.36 log_2_fold increase, has been previously associated with photomorphogenesis and flowering. This TF mediates cytokinin responses and modulates the auxin feedback system, emphasizing its potential significance in the drought response^31,32^. In addition, the *Anthocyanin regulatory C1 protein* (BaRT2v18chr7HG342790), belonging to the MYB family, was upregulated by 7.51 log_2_fold, signifying its potential role in pigment regulation and stress responses. The ERF family is represented by the *Ethylene-responsive transcription factor 11* (ERF11; BaRT2v18chr5HG235500). ERF11 was upregulated in leaves with a 6.78 log_2_fold increase, highlighting its already-known significance in the stress responses^33^. Lastly, the WRKY family, specifically WRKY40 (BaRT2v18chr4HG197280), with a 3.04 log_2_fold upregulation, has been linked to ABA signaling and defence responses^34,35^.

Among DAS genes in leaves, the C2H2 (4 genes), C3H (4 genes), and G2-like (3 genes) transcription factor families emerged as key regulators (Fig. 3B). Notably, *PROTEIN INDETERMINATE-DOMAIN 5* (*IDD5*, BaRT2v18chr2HG095740), representing the C2H2 family, displayed significant splicing variation, suggesting potential roles in stress responses and developmental cues.

In the roots, transcriptional changes underscored the prominence of the ERF (8 genes), bZIP (8), MYB (6), C2H2 (5), and bHLH (5) TF families. We observed that *ERF11* (BaRT2v18chr5HG235500) demonstrated consistent significance in leaves and roots. Within the bZIP family, a noteworthy gene encoding a protein potentially associated with the action of ABSCISIC ACID-INSENSITIVE 5 (BaRT2v18chr5HG259930) is upregulated. Other significant genes included *MYB-related transcription factor* (BaRT2v18chr4HG197580) and zinc finger protein *WIP6* (BaRT2v18chr3HG156400; C2H2 family). Further analysis has revealed that the homolog of BaRT2v18chr4HG197580 in Arabidopsis encodes a regulator of anthocyanin accumulation under salinity stress^36^. In the case of *WIP6* identified in our study, its homolog in Arabidopsis is involved in cell fate determination during primary root development^37^. DAS events in roots highlighted the FAR1 (5 genes), MYB_related (5), and NAC (5) TF families.

### Isoform dynamics in barley roots under drought

**Fig. 4:**
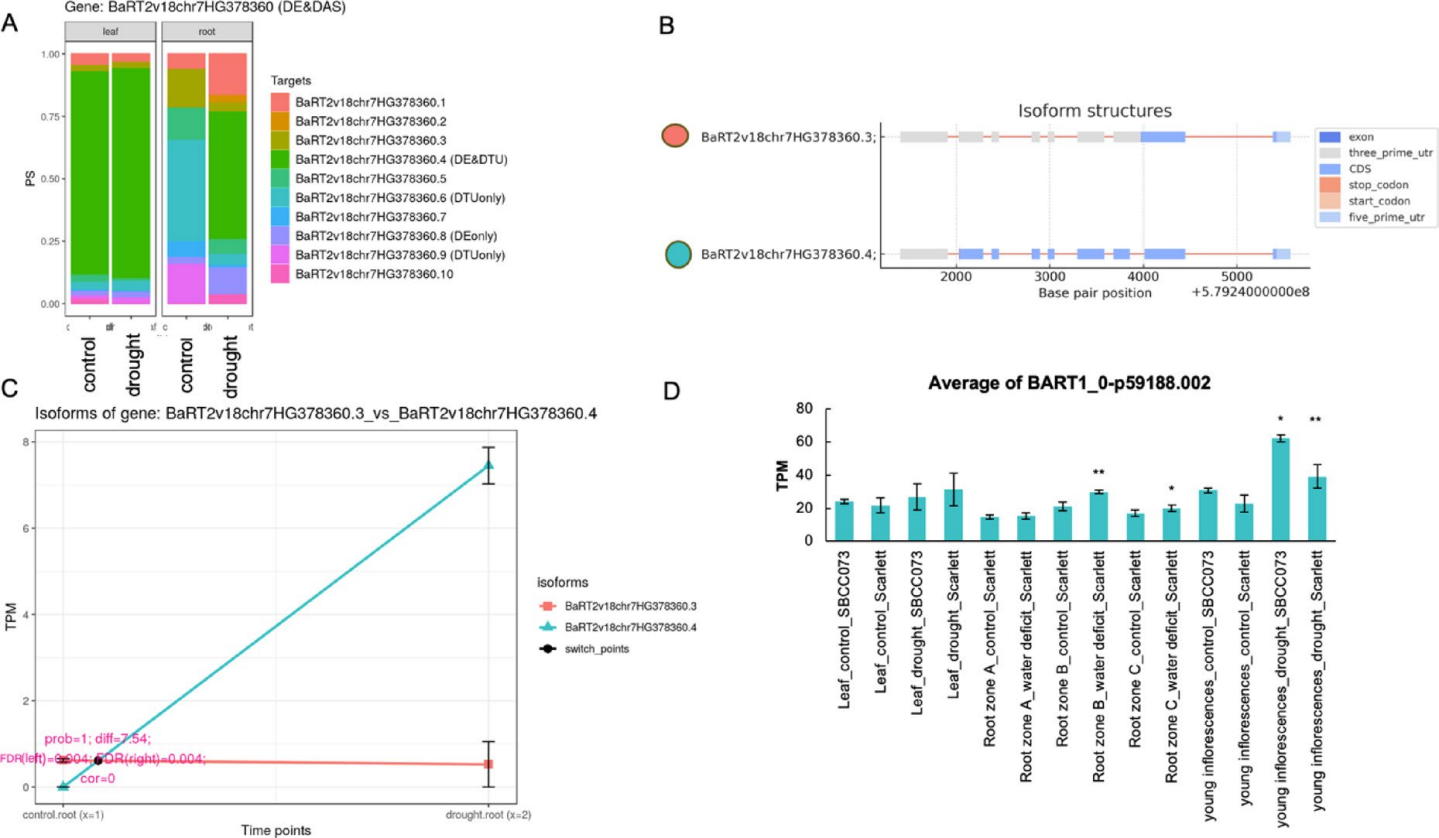
Characterization and differential expression of BaRT2v18chr7HG378360 (E3 ubiquitin-protein ligase MARCH6) isoforms in response to drought stress. (**A**) Proportionately spliced (PS) variants of BaRT2v18chr7HG378360 isoforms in leaf and root under control and drought; **(B**) Structure of BaRT2v18chr7HG378360.4 (DE&DTU) and BaRT2v18chr7HG378360.3 isoforms; **(C)** Expression of BaRT2v18chr7HG378360.4 and BaRT2v18chr7HG378360.3 isoforms in roots under control and drought stress; (**D**) The expression of BaRT2v18chr7HG378360.4 (corresponding to BART1_0-p59188.002 in the previous version of Barley RTD) under drought (based on data downloaded from the EoRNA database, Zone A corresponds to the meristematic region, Zone B to the elongation zone, and Zone C to the maturation zone^86^. Asterisks indicate statistically significant differences between treatment and control based on a t-test: *p* < 0.05 (*), *p* < 0.01 (**), and *p* < 0.001 (***).

**Fig. 5:**
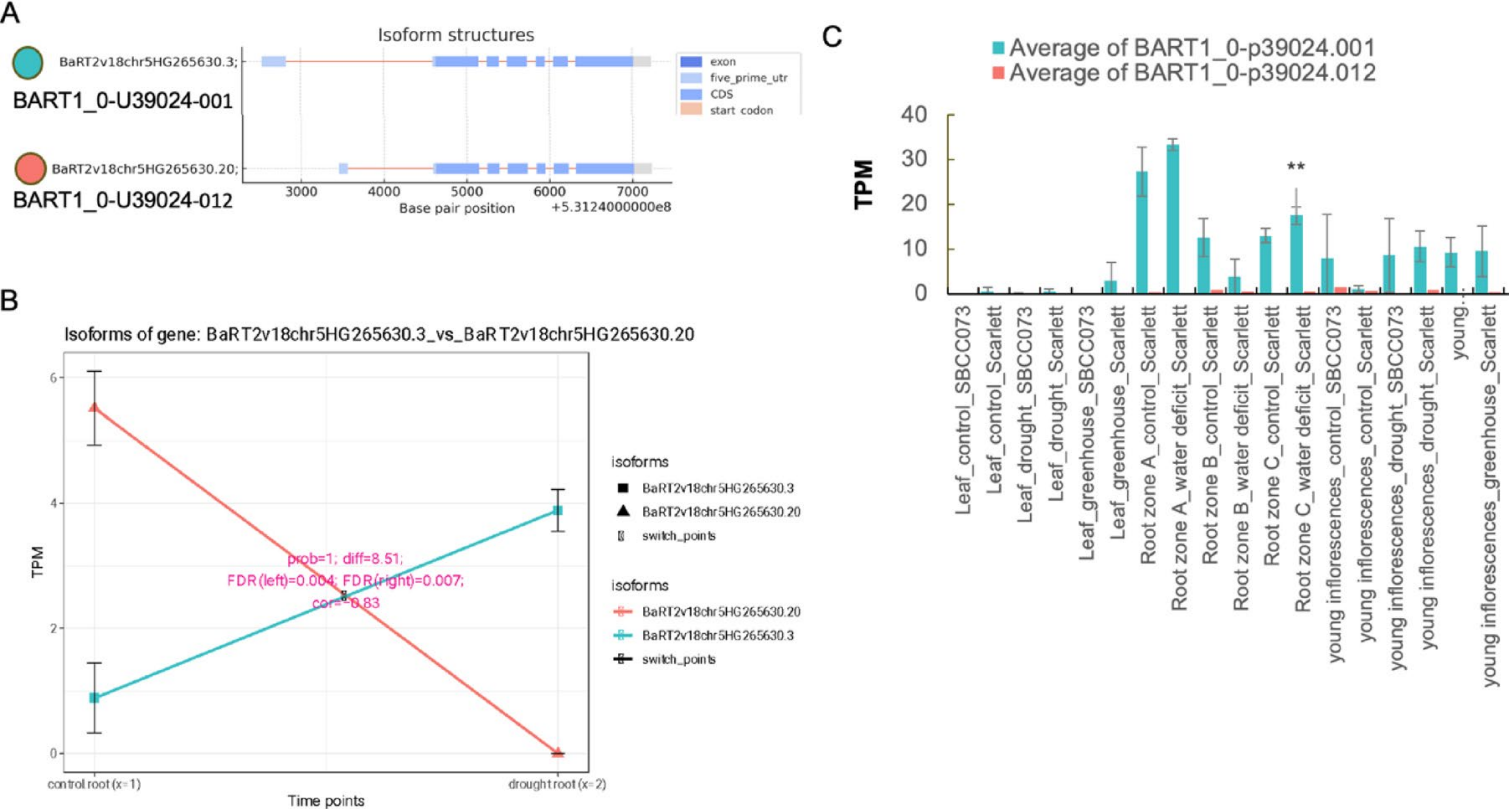
Isoform dynamics and differential expression of BaRT2v18chr5HG265630 in response to drought stress. (**A**) Comparison of isoform structures: BaRT2v18chr5HG265630.3 and BaRT2v18chr5HG265630.20, **(B)** Expression shift of BaRT2v18chr5HG265630 isoforms under control and drought conditions; **(C)** Transcript expression under drought (based on data downloaded from EoRNA database; Zone A corresponds to the meristematic region, Zone B to the elongation zone, and Zone C to the maturation zone^86^. Asterisks indicate statistically significant differences between treatment and control based on a t-test: *p* < 0.05 (*), *p* < 0.01 (**), and *p* < 0.001 (***).

Isoform switching (IS) allows fine-tuned cellular responses and plays a pivotal role in physiological adaptations and stress conditions. In this study, we investigated IS in barley roots under drought stress. We focused on the roots because their drought response remains elusive at the molecular level. The BaRT2v18chr7HG378360 gene, which exhibits an isoform switch, encodes a RING-CH-type domain-containing protein that plays a role in mRNA turnover and stability, and acts as an E3 ubiquitin-protein ligase MARCH 6 (Fig. 4). We identified two isoforms of this gene: BaRT2v18chr7HG378360.3, an unproductive variant with very low expression under both control and drought conditions, and BaRT2v18chr7HG378360.4 (DE&DTU) (Fig. 4A, B), which encodes a fully productive protein. After drought stress, the isoform BaRT2v18chr7HG378360.4 was highly expressed specifically in roots (Fig. 4C). Further investigation using the EoRNA database^38^ showed that drought stress specifically upregulated this isoform in root zones B (25–50% of total root length) and C (50–100% of total root length), as well as in young inflorescences of the Scarlett cultivar (Fig. 4D). The dynamics of isoform switching in roots under drought conditions are further exemplified by the BaRT2v18chr5HG265630 gene, which encodes the Beta-glucosidase BoGH3B. We identified two isoforms of this gene: BaRT2v18chr5HG265630.3, which shows low expression under control conditions but is elevated under drought conditions, encoding a complete protein; and BaRT2v18chr5HG265630.20, which exhibits high expression under control conditions but diminishes during drought, leading to a truncated protein (Fig. 5 AB). Transcript expression data from the EoRNA database revealed the corresponding transcripts for these isoforms: BART1_0-U39024-001 for BaRT2v18chr5HG265630.3 and BART1_0-U39024-012 for BaRT2v18chr5HG265630.20. Intriguingly, BART1_0-U39024-001 was upregulated under drought conditions, specifically in basal root zone C of the Scarlett cultivar (Fig. 5C), supporting our results.

### Alterations in hormonal profiles of barley under drought conditions

**Fig. 6:**
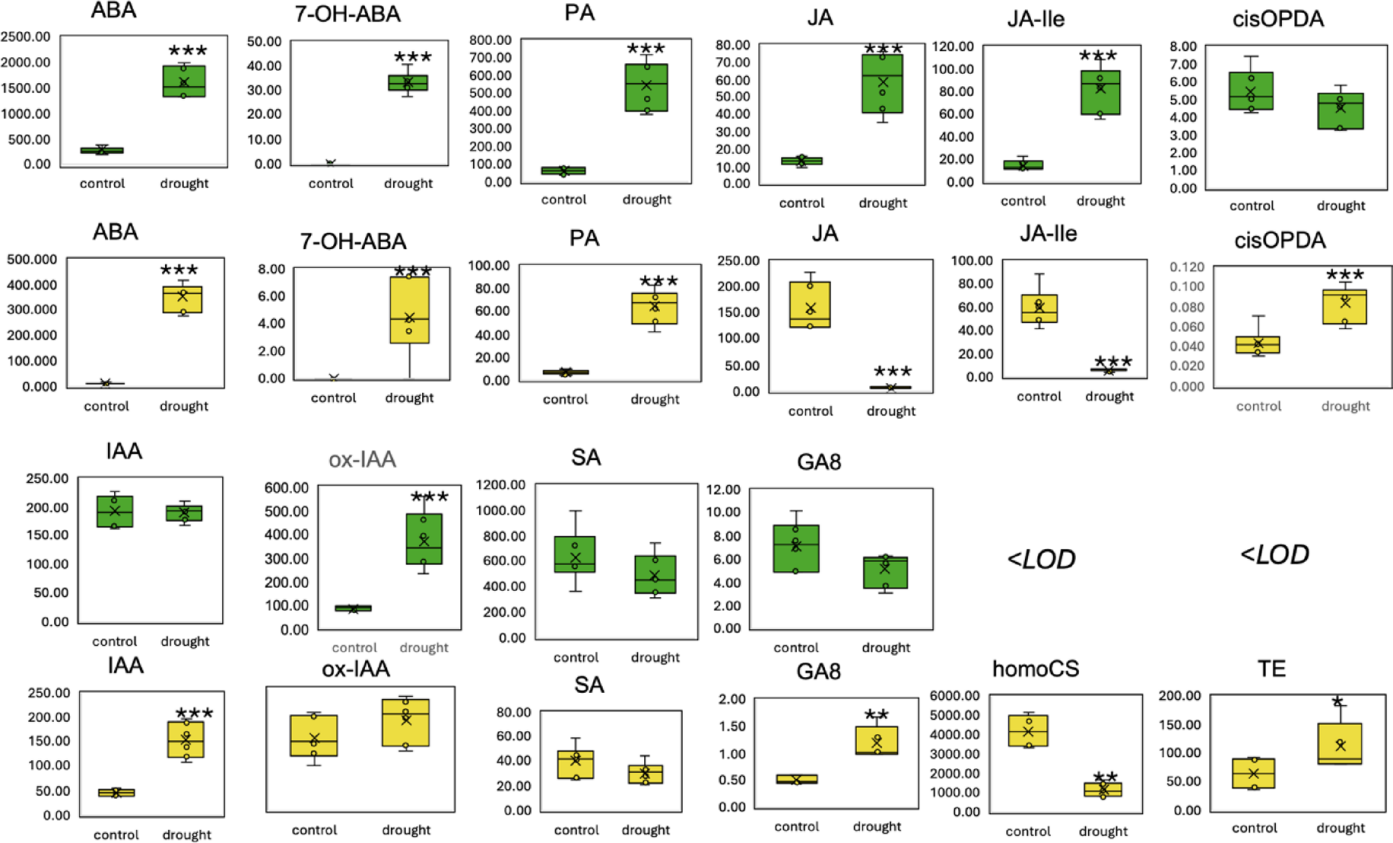
Comparative analysis of key phytohormones in barley leaf (green) and root (yellow) under drought. The green designates values in leaf, while yellow for roots. ABA – abscisic acid, 7-OH-ABA − 7’-Hydroxy ABA, PA – phaseic acid, JA – jasmonic acid, JA-Ille - Jasmonoyl isoleucine, cis-OPDA − 12-Oxo-Phytodienoic Acid, IAA - Indole-3-acetic acid, ox-IAA − 2-oxindole-3-acetic acid, SA – salicylic acid, GA8 – gibberellin A8, homoCS – homocastasteron, TE – teasteron. < LOD – under detection limit; all data in pmol/g FW. Asterisks indicate statistically significant differences between treatment and control based on a t-test: *p* < 0.05 (*), *p* < 0.01 (**), and *p* < 0.001 (***).

To elucidate the hormonal adaptations of barley in response to drought, we profiled the hormone content in both leaves and roots under control and drought treatments (25DAP) (Fig. 6). Abscisic Acid (ABA) levels were increased in the leaves in response to drought. This elevation was even more evident in the roots. We also found a marked increase in ABA derivatives, such as 7-OH-ABA, Phaseic Acid (PA), in both leaves and roots. Interestingly, the jasmonates showed distinct organ-specific behaviors. The root concentrations of Jasmonic Acid (JA) and its derivative JA-Ile decreased dramatically, whereas in stark contrast, JA and JA-Ile levels were amplified in the leaves. Auxin levels show significant differences between leaf and root. While root auxins (IAA) increased, hinting at an attempt to optimize root growth for deeper soil moisture acquisition, leaf IAA concentrations did not change (Fig. 6). Interestingly, the elevation of auxin-related compounds, such as ox-IAA, in the leaves underlined a harmonized auxin-mediated response across barley organs. Out of analyzed gibberellins, only GA8 showed significant content shift. Notably, GA8 levels in the leaves decreased whereas the significant surge in gibberellin form GA8 in roots was observed (Fig. 6).

To investigate if barley employs orchestrated interplay between hormonal dynamics and transcriptional modulation in response to drought stress, we analyzed our transcriptomic data and filtered them against GO terms, indicating the involvement of genes in phytohormonal pathways. We obtained a dataset of 217 and 117 DE hormone-related genes in leaves and roots, respectively (Supplementary Table S5). Significant transcriptional changes mirrored elevated ABA concentrations in roots and leaves. Five of the seven genes related to ABA that showed differential expression were upregulated in roots. Notably, genes encoding late embryogenesis abundant protein 3 (BaRT2v18chr1HG040290 and BaRT2v18chr1HG040250) were markedly upregulated, emphasizing the central role of ABA in drought adaptation. Eight of the 14 genes associated with ABA in leaves were upregulated. Downregulated ABA-related genes were mostly encoded ABA receptors, i.e., Abscisic acid receptor PYR1 (BaRT2v18chr2HG092820) or Abscisic acid receptor PYL4 (BaRT2v18chr2HG056780). JA dynamics exhibit organ-specific nuances. In the roots, where JA content decreased, most of the 13 JA-associated genes were downregulated. Conversely, in leaves where JA content increased, seven of the 16 JA-related genes were upregulated. Among them, the 23 kDa jasmonate-induced protein (BaRT2v18chrUnG390490, BaRT2v18chrUnG392100) and jasmonate-induced protein (BaRT2v18chr1HG000490) showed pronounced upregulation.

Auxin dynamics displayed contrasting behaviors between the roots and leaves. In the roots, upregulation was observed in seven of the ten auxin-associated genes, reflecting increased IAA content. Genes pivotal for auxin transport and signaling, such as *E3 ubiquitin-protein ligase UBR4* (BaRT2v18chr5HG242140) or *Auxin-responsive protein IAA14* (BaRT2v18chr5HG271040), showed pronounced upregulation. In leaves, however, modulation was observed in the expression of genes such as the *Auxin efflux carrier component* (BaRT2v18chr3HG162730), partially aligning with the stable IAA levels. Gibberellin-associated genes in the leaves also exhibit adaptive responses to drought. Seven of the 12 genes were upregulated, including *Gibberellin 2-beta-dioxygenase* (BaRT2v18chr2HG092430) and *Gibberellin receptor GID1* (BaRT2v18chr1HG028980).

### Barley rhizosphere microbial analyses under drought stress

**Fig. 7:**
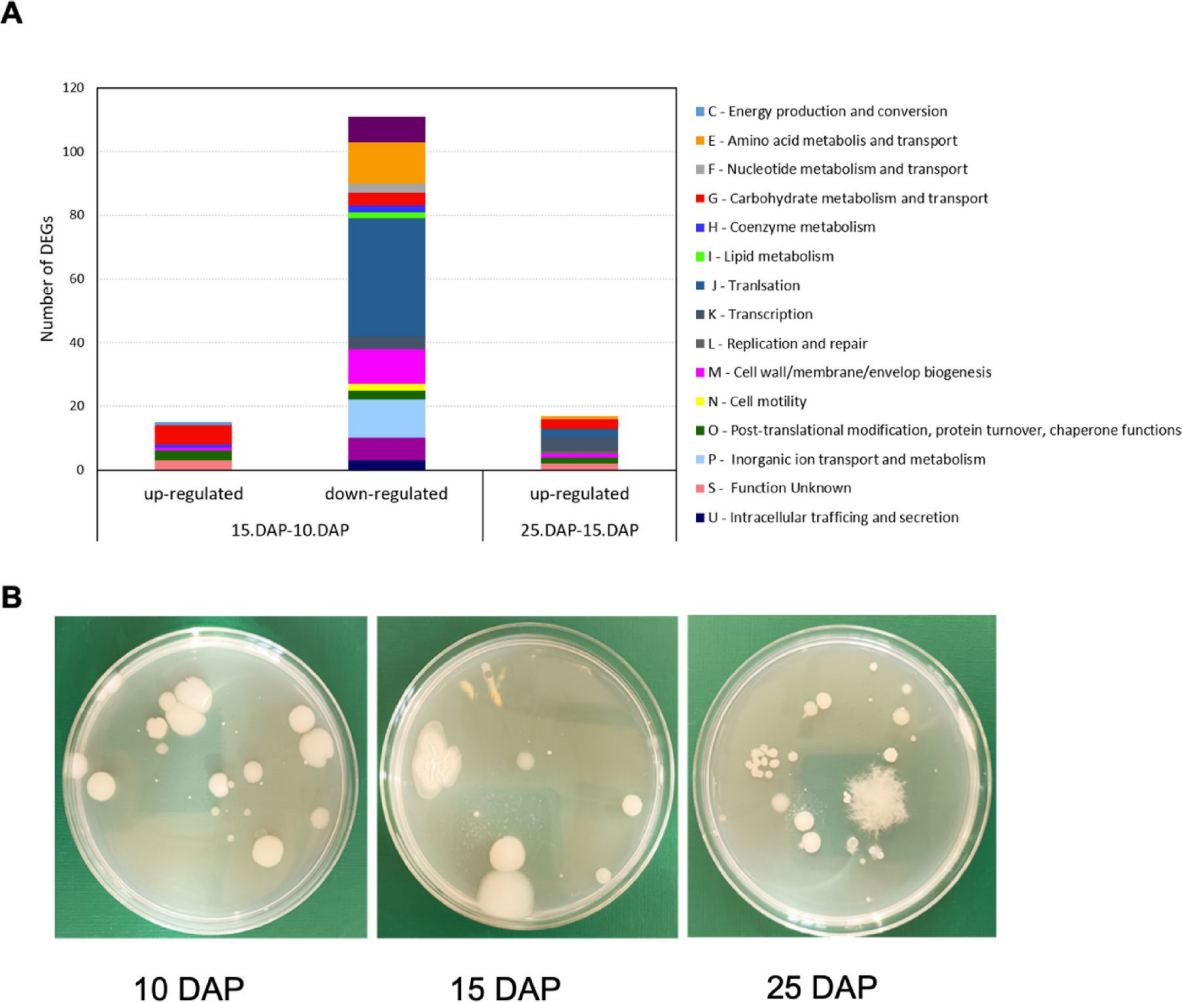
Rhizosphere analysis. (**A**) Comparative analysis of bacterial differentially expressed genes (DEGs) functionally classified to COGs categories within selected contrast groups. (**B**) Bacterial colonies on LB medium isolated from rhizosphere soil at selected time points of the drought experiment (the same dilutions were applied).

Next, a limited assessment of the barley rhizosphere microbial community under drought stress was also undertaken. We retrieved 1,079,732,590 total reads that were assembled into 13,194 transcripts in the analyzed soils. To compare changes in the expression of microbial genes between soils taken from different time points of the experiment, three contrasting groups were set: (i) 15 DAP vs. 10 DAP; (ii) 25 DAP vs. 15 DAP; and (iii) 25 DAP vs. 10 DAP. The most distinct differences in the levels of bacterial gene expression were observed already when the onset of drought versus optimal water condition samples were compared. At 15 DAP, 126 genes showed altered expression. These genes were functionally categorized based on the COG (Clusters of Orthologous Groups) database (capital letters next to colored squares in Fig. 7A and square brackets in the text). Only 15 genes were upregulated, whereas the remaining 111 genes were downregulated. Among the upregulated genes encoding heat shock proteins (*Heat-induced stress protein YflT* (Gene2040) [S], *Small heat shock protein* (HSP20) family (Gene1520) [O], and *Heat shock 70 kDa protein* (HSP70) family (Gene 4250) [O], genes encoding enzymes responsible for carbon metabolism [G] were detected. Downregulated genes were predominantly associated with transcriptional [K] and translational [J] processes, especially genes encoding proteins that build individual subunits of bacterial ribosomes (Supplementary Table S6). When comparing the expression levels of bacterial genes in samples from 25 DAP with those from 15 DAP, only 17 genes were upregulated and no downregulated genes were identified. Among the upregulated genes are those encoding proteins that are part of the chaperone system involved in the cell regeneration process after heat-induced damage (Gene0656) [O]. Genes encoding transcription [K] and translation [J] factors were also identified (Fig. 7A). It is noteworthy that these processes were associated with bacteria belonging mainly to Actinobacteria, Firmicutes, and Chloroflexi, which are known for their ability to adapt to and tolerate high temperatures and survive in soil under periodic water shortages (Supplementary Table S7). Surprisingly, we could not detect significant DEGs when 25DAP versus 10 DAP were analyzed.

Despite high read counts, metatranscriptomics yielded few transcripts^39–41^. This suggested a low number of bacteria in the tested soil. To confirm this hypothesis, additional analyses, including estimation of the culturable fraction of bacteria and PLFA identification, were performed. The total number of colony-forming units (CFU) isolated from the tested soils was slightly lower than 1 × 106 g-1 dry weight (d.w.) of soil, regardless of the water content. Bacterial colonies grown on Luria-Bertani medium were characterized by low morphological diversity, and 95% of the isolates were recognized as spore-forming Gram-positive strains (Fig. 7B). Additionally, only 5% of the colonies were assigned to Actinobacteria, and no Gram-negative bacteria were isolated from the tested soils on the LB medium. The low abundance of living microorganisms in the soil samples was also confirmed by the PLFA analysis (Table 1). All tested soils showed a dominance of branched fatty acids, which are indicators of Gram-positive bacteria. However, their amount was low and did not exceed 1.5 nmol PLFA g-1 d.w. of soil.

**Table 1:** Amount of marker PLFAs values in the soils during the experimental period (nmol PLFA g^-1^ d.w. of soil).

Sample	Gram-negative	Gram-positive	*Actinobacteria*	Fungi
10 DAS	0.154 ± 0.011^b^	0.547 ± 0.063^b^	0.000 ± 0.000^b^	0.087 ± 0.034^a^
15 DAS	0.500 ± 0.066^a^	1.442 ± 0.207^a^	0.306 ± 0.039^a^	0.055 ± 0.012^a^
25 DAS	0.344 ± 0.107^a^	1.189 ± 0.255^a^	0.227 ± 0.052^a^	0.045 ± 0.02^a^

Different letters (within each group) indicate significant differences (*P* < 0.05, Tukey test); ± Stand. Dev. of three independent experiments

## Discussion

We investigated adaptive strategies and observed marked differences in how leaf and root respond at the molecular level. Our results showed that in leaf genes associated with photosynthesis, particularly those linked to light reactions and harvesting, were predominantly downregulated under drought conditions. This downregulation, especially of photosynthesis-related processes (GO:0019684), suggests a strategy for conserving energy and resources in the face of reduced water availability. Drought-induced downregulation of genes involved in chlorophyll metabolism, photosynthesis, carbon metabolism, and energy conversion has been already observed in maize and tobacco^42,43^.

In contrast, upregulated genes in leaves were enriched in categories signifying responses to water (GO:0009415) and water deprivation (GO:0009414). This indicates an active molecular effort to manage water loss and optimize water usage. Genes annotated as overrepresented in these categories are homologs of well-known drought regulators, that is, transcription factors from the NAC, WRKY, and MYB TF families.

Roots are the primary water-absorbing organs. Our data revealed a significant downregulation of genes related to DNA integration (GO:0015074) and RNA-templated DNA biosynthesis (GO:0006278). This suggests a potential reduction in cellular growth or division, suggesting a root strategy to curtail energy-intensive processes and redirect resources to essential survival mechanisms under water-deficit conditions. Under drought conditions, limited water and nutrient uptake reduce root growth and development^44^. Furthermore, water scarcity can induce root structural changes. Drought promotes a higher ratio of large cortical cells in roots to decrease energy costs^45,46^ Simultaneously, anatomical changes such as suberin and lignin deposition are observed^46^.

Conversely, the upregulated genes in the roots were enriched in metabolic processes. Particularly notable were those associated with polyamine metabolism (GO:0006595) and biosynthesis (GO:0006596). Because polyamines play a role in abiotic stress tolerance, their upregulation underscores the active molecular mechanisms of roots that enhance drought adaptation. Furthermore, the prominence of genes related to carbohydrate metabolism, particularly those associated with sucrose metabolism (GO:0005985), suggests potential shifts in energy storage or osmotic balance strategies during drought stress. Our comprehensive analysis of barley drought response emphasizes that the leaf pivots towards conserving energy and enhancing water-use efficiency, whereas the root engages in metabolic adjustments, emphasizing survival and adaptive growth.

Alternative splicing (AS) is a pivotal regulatory mechanism in barley adaptation to drought^10^. Our data indicated that 2,362 (8.6%) genes underwent differential alternative splicing under drought. This flexibility in producing multiple protein variants from a single gene or in controlling the turnover and level of protein-coding transcripts is crucial under stress conditions. One of the adaptation mechanism based on AS is isoform switching which enables plants to rapidly modify their protein landscape. The gene BaRT2v18chr7HG378360 showcased a shift in isoforms that are predicted to modulate the functionality of an E3 ubiquitin-protein ligase, which plays a role in protein degradation and signaling. Such modulation could allow barley to regulate protein turnover rates, ensuring that only the most drought-adaptive proteins are synthesized and retained under water-deficit conditions. Ubiquitin E3 ligases play a significant role in several vital processes such as enduring stress, defense against viruses, fighting diseases, controlling cell mortality, and facilitating the release of amino acids^47–49^.

Genes encoding drought regulators such as transcription factors are often targets of AS, and different isoforms can exert different effects on the plant response to drought. Some isoforms are effective drought regulators, whereas others can be expressed under optimal water supply conditions and do not affect plant growth^50,51^. By producing different protein variants of the same gene, barley can tailor its molecular response to specific drought conditions. This mechanism allows subtle adjustments in protein function without necessitating large-scale transcriptional changes^52,53^.

The resilience of plants to drought stress is related to their hormonal background. Abscisic Acid (ABA) is essential for plant adaptation to water scarcity^54,55^. Our data underline the significance of ABA, revealing a 30-fold increase in leaf ABA concentration under drought stress. Reflecting ABA-dependent response, genes such as *Late embryogenesis abundant protein 3* (BaRT2v18chr1HG040290 and BaRT2v18chr1HG040250), which encode proteins that protect cells against the consequences of water shortage, are activated by ABA-dependent bZIP transcription factors^56,57^. Conversely, downregulated ABA-related genes, such as *Abscisic acid receptor PYR1* (BaRT2v18chr2HG092820) and *Abscisic acid receptor PYL4* (BaRT2v18chr2HG056780), further hint at the complexity of the ABA-mediated response. Downregulation of genes encoding ABA receptors in barley under drought conditions has been observed in several studies^56,58,59^. This is likely a part of the fine-tuning mechanism within core ABA signaling, preventing over-activation of the ABA pathway in response to stress.

Very different organ-specific responses between leaves and roots concentrations of Jasmonic Acid (JA) may indicate a strategic shift in resource allocation, possibly conserving energy and nutrients for other vital processes under water deficit conditions. JA root signals can regulate shoot responses to drought^60^. Therefore, the root-to-shoot allocation of JA could be part of the JA-mediated regulation of drought response. Notably, the upregulation of JA-associated genes, such as the *23 kDa jasmonate-induced protein* (BaRT2v18chrUnG390490, BaRT2v18chrUnG392100), provides molecular evidence of enhanced JA-mediated defense or regulatory roles under drought. As drought can render plants more vulnerable to opportunistic pathogens, this increase in JA might be a proactive measure to boost leaf defense systems. The activated JA response is usually correlated with better tolerance to drought. JA is involved in ROS scavenging, stomatal closure, and modulation of ABA signaling^61–63^.

Auxins are involved in response to drought by promoting the growth of primary and lateral roots to access water in deeper soil^64^ and by inhibiting leaf development^65^. Auxins showed elevated root levels of Indole-3-acetic acid (IAA), which may be an adaptive strategy driving the plant to prioritize root growth in search of deeper water reserves. This is further supported by the upregulation of genes pivotal for auxin transport and signaling, such as *E3 ubiquitin-protein ligase UBR4* (BaRT2v18chr5HG242140) and *Auxin-responsive protein IAA14* (BaRT2v18chr5HG271040). Conversely, the reduction in leaf IAA, mirrored by modulated expression of genes such as *Auxin efflux carrier component* (BaRT2v18chr3HG162730), could be an energy-conservation tactic, curtailing unnecessary growth and metabolic activities under stressful conditions.

Comparison of transcriptomes response to drought of ‘Sebastian’ and drought tolerant barley cultivars, ‘Otis’, ‘Maresi’ and breeding line ‘CamB’, enabled to highlight some common elements^66,67^. Although experiment setups for ‘Sebastian’ and these barley cultivars/breeding line were different, it allowed to elucidate conserved parts of molecular response to drought. Some GO biological processes were shared between ‘Sebastian’ and ‘Otis’ in response to drought such as response to water or sucrose metabolism. Moreover, drought-treated leaves of all cultivars/breeding line exhibited downregulation of processes related to chlorophyll biosynthesis/photosynthesis. On the other side, we did not found lipid metabolism in overrepresentated GO terms in ‘Sebastian’ as it was observed in ‘Otis’, ‘Maresi’ and ‘CamB’ under drought. Insight into DEGs also revealed some common parts of drought response. Differentiated regulation of genes encoding ABCG transporters which play important role in phytohormonal transport, including ABA, was found in ‘Sebastian, ‘Otis’, ‘Maresi’ and ‘CamB’. Furthermore, elevated expression of genes encoding key ABA biosynthesis enzyme, *9-cis-epoxycarotenoid dioxygenase* (*NCED*), was observed in roots of ‘CamB’ and ‘Sebastian’. On the other side, downregulation of phosphorylation related genes was found in drought treated roots of ‘Sebastian’ and ‘CamB’, whereas in leaves downregulation of genes involved in BR signaling was shared response to drought of ‘Sebastian’, ‘Maresi’ and ‘CamB’. It is noteworthy that some exclusive elements of drought response were also identified in these cultivars, e.g. only ‘Otis’ showed increased expression of genes encoding homologs of *Glossy1* and *Eceriferum1*, genes involved in long chain fatty acid biosynthesis for wax production in leaves, whereas ‘Maresi’ exhibited elevated expression of genes responsible for fructose metabolism in roots.

In rhizosphere, plants and their associated microbes form mutualistic relationships that influence health, growth, and adaptive strategies^68^. Our metatranscriptomic analysis of the barley rhizosphere under drought conditions, supported by the analysis of bacterial culturable fraction (plating method) and total microbial biomass (PLFA), provides some understanding of plant and microbe interactions. The applied soil served as a harsh environment for microorganisms, even under optimal water conditions, as observed for the 10 DAP samples and pronounced under water deficit conditions. This suggests the activation of bacterial defence response rather than the activity of processes supporting plants during drought. The highly unfavorable conditions for the development of microorganisms in the tested soil are also evidenced by the dominance of spore-producing bacterial strains, which ensure the survival of adverse environmental conditions^69^. This also explains the low microbial activity expressed as the number of transcripts isolated from the tested soils. The number of bacterial CFU in the rhizosphere of plants/crops in arable soil can reach values even 3 orders of magnitude higher than that observed in our soil^70^. The amount of microbial biomass in our soils, expressed in nmol PLFA, did not exceed 2.3 g^−1^ d.w of soil. In contrast^71^ estimated the total microbial biomass in arable soil to be between 35 and 40 nmol PLFA g^−1^ d.w. of soil. This indicated a low number of living microbial cells in the tested rhizospheric soil during the entire drought experiment.

We suspect that changes in the expression of the selected bacterial genes are related to the groups of bacteria that are most resistant to drought stress. This group includes the phylum taxonomically affiliated as *Chloroflexi*^72^, which was detected in our study. In turn, the dominant cultivable fraction of bacteria from the genus *Bacillus* is known to produce spores under unfavorable environmental conditions, which might artificially overstate their number, as determined by the microbiological media. Similarly, soil over-drying favors the *Actinobacteria* group, whereas other drought-sensitive bacteria are eliminated^72^.

In conclusion, our integrated transcriptomic and hormonomic analyses demonstrate that drought induces profound, organ-specific reprogramming in barley. In leaves, the downregulation of photosynthesis-related genes and the upregulation of water response and ABA signaling pathways suggest a strategic shift toward stress mitigation. Conversely, roots exhibit a broader spectrum of transcriptional changes, including extensive alternative splicing and isoform switching, which likely fine-tune cellular functions to optimize water uptake and maintain growth. Hormonal profiling further revealed that ABA accumulation, particularly in roots, along with organ-specific modulation of jasmonates and auxins, plays a central role in orchestrating these adaptive responses. Although our assessment of the rhizosphere microbial community was limited by low transcript abundance and a predominance of Gńżram-positive, spore-forming bacteria, the data imply that intrinsic plant mechanisms are the primary drivers of drought adaptation under the conditions tested. Collectively, these findings provide a comprehensive framework for understanding barley’s multilayered response to drought and offer valuable insights for developing more resilient crop varieties in the face of climate change.

## Materials and methods

### Drought stress experiment

The plant material used in this study was the spring barley (*Hordeum vulgare* (L.)) cultivar ‘Sebastian’. Sebastian is a two-row spring barley cultivar developed by Sejet Planteforædling I/C (Denmark). Sebastian combines high yield potential (up to 10 t/ha) with drought tolerance, resistance to powdery mildew and helminthosporia, and stable agronomic performance. Drought stress was induced using a previously described method^56^. Briefly, we used boxes filled with a soil mixture of sandy loam and sand (7:2) derived from Pulawy (Poland) with specific physicochemical properties and nutrient medium (Supplementary Material S8). Plants could easily access water at a volumetric water content (vwc) of 14% in the soil, whereas the water deficit condition was 1.5%. At 14% vwc, leaf water content was approximately 100%. The soil moisture was checked daily using a time-domain reflectometer (TDR) EasyTest. For the first 10 days after sowing, plants were grown in a greenhouse with optimal water (14% vwc), 20/18 °C day/night temperature, 16/8 h photoperiod, and 420 µmol m^−2^ s^−1^ PAR from fluorescent lamps. Irrigation was then withheld until the soil moisture reached 3% on the 15 th day (15DAP, Days After Planting), marking the start of drought. Plants were then moved to a chamber under a 25 °C/20 °C day/night regime, 16/8 h photoperiod, and 420 µmol m^−2^ s^−1^ light for 10 days (until 25 DAP), indicating prolonged drought at 1.5% vwc. Control plants had optimal water availability and were grown simultaneously. The phenotype of plants analyzed on 10, 15 and 25 DAP is presented on the Supplementary Figure S1. Soil tightly adhered to the roots after removal of plants from the pots (rhizosphere) was collected by root shaking at the following time points: 10, 15, and 25 DAP. The second leaf and root were harvested 25 days after planting (DAP) (for both treatments, control growing under optimal conditions and after 10-day-long drought stress) for RNA extraction. All analyses were performed on the second leaf, which was present when the drought started, and each genotype was analyzed in three replicates, with one box of 15 plants per genotype as one replicate.

### Transcriptomics analysis

Leaf and root tissues were collected in three replicates, each consisting of three fragments of independent leaves/roots. Entire root systems were collected, gently washed with distilled water to remove adhering soil, blotted dry, and immediately frozen. Root samples were later homogenized as mixtures of different zones to avoid bias in gene expression from specific regions. For shoots, the central portion of the second fully expanded leaf was sampled at 10 days post-germination, ensuring developmental consistency across plants. Each biological replicate comprised tissue pooled from 3 to 4 independent plants. RNA was extracted using a mirVana RNA isolation kit (Thermo Fisher Scientific), according to the manufacturer’s instructions. Transcriptome deep sequencing was performed using the short-read Illumina sequencing, 2 × 150 bp PE reads. RNA-seq reads were aligned to the barley reference transcriptome BaRTv2.18^17^ using the Kallisto tool for accurate transcript quantification^73^. Differential expression analyses at the gene, transcript, and alternative splicing levels were performed using 3D RNA-seq application (https://3drnaseq.hutton.ac.uk/app_direct/3DRNAseq/) and by setting contrast groups of drought against control in leaf and root tissues. Differentially expressed genes (DEGs) and transcripts (DETs) were determined by FDR adjusted p-value < 0.01 and absolute value of log_2_-fold-change $\:\ge\:$ 2. Differential alternative expressed (DAS) genes and differential transcript usage (DTU) transcripts were determined by FDR adjusted p-value < 0.01 and transcript ratio change $\:\ge\:10\%$. The 3D RNA-seq application also pinpointed transcripts that showed significant abundance switches between drought and control. Isoform switches were identified using the 3D RNA-seq pipeline based on the iso-kTSP method^74^, which detects switches between alternatively spliced isoforms in pairwise contrast groups. Significant switches were determined by the probability of switch across replicates, the sum of average expression differences before and after the switch, and Benjamini-Hochberg adjusted p-values. Gene Ontology enrichment analysis of genes with significant changes and isoform switches was conducted using the TopGO R package (https://bioconductor.org/packages/topGO/). The significance of enrichment was derived from an FDR of < 0.05. We used full set of DE genes (FDR < 0.05), and DAS genes (ΔPSI >10%, FDR < 0.05) as input for GO enrichment analysis. The PlantRegMap tool was used to predict transcription factors (TFs)^75^ encoded by DEGs and DAS identified. TF annotation was performed using the updated family assignment criteria implemented in PlantTFDB v5.0 (https://planttfdb.gao-lab.org/prediction.php), which considers DNA-binding domain, auxiliary domain, and the presence of forbidden domains. Only those proteins containing characteristic TF domains and lacking forbidden domains (indicative of non-transcriptional regulatory functions) were retained as bona fide TFs.

### Metatranscriptomics analysis

Rhizosphere samples were collected in three replicates. RNA was extracted using the RNeasy PowerSoil Total RNA Kit (Qiagen, Germantown, MD, USA) according to the manufacturer’s instructions. Ribosomal RNA depletion was performed using the NEBNext rRNA Depletion Kit (Bacteria) (New England BioLabs, Germany), and the library was constructed using TruSeq Stranded Total RNA (Illumina, San Diego, CA, USA). Libraries were sequenced using Illumina NovaSeq 6000 (2 × 150 bp, paired-end, Illumina, San Diego, CA, USA). Data were analyzed using ATLAS pipelines (a workflow for assembly, annotation, and genetic binning of metagenome sequence data, https://github.com/metagenome-atlas/atlas)^76^. Briefly, quality control of raw sequences, error correction based on k-mer coverage, and paired-end read merging were performed using utilities in the BBTools suite, and metaSPAdes were used for *de novo* assembly^77^. For annotation, open reading frames (ORFs) were predicted using Prodigal, and the translated gene products were then clustered using Linclust to generate non-redundant gene and protein catalogs, which were mapped to the eggNOG catalog v5.0^78^. Differential abundance analysis was performed using the MaAsLin 2 (Microbiome Multivariable Associations with Linear Models) package (Bioinductor), a multi-model approach for conducting multivariable association testing in microbiome datasets^79^ employing the R software. Genes with a P-value (P-adj) < 0.05, and an absolute value of coefficient > 2.0, were considered differentially expressed. Gene Ontology (GO) enrichment and KEGG enrichment were conducted using TopGO and clusterProfiler^80^ packages to identify the biological functions and pathways affected by differentially expressed genes (DEGs), respectively.

### Phospholipid fatty acid (PLFA) analysis

PLFAs with a carbon chain length between 9 and 20, indicate the presence of living microorganisms in the soil. They build microbial cell membranes and their amount and composition are strictly correlated with microbial abundance and biodiversity. PLFAs were isolated from 10 g of soil, as described by^81^. Fatty acid methyl esters were separated and identified, as described by^82^. The bacterial biomass of Gram-negative and Gram-positive bacteria was calculated as the sum of 16:1 ⍵9c, 16:1 ⍵7c, 17:0 cyclo, 18:1 ⍵7c, 19:0 cyclo, 19:0 cyclo ⍵8c and 15:0 iso, 15:0 anteiso, 16:0 iso, 17:0 iso, and 17:0 anteiso, respectively. Methylated 17:0 10-methyl and 18:0 10-methyl were used to determine the biomass of Actinomycetes, and polyunsaturated 18:2 ⍵6,9c and 18:3 ⍵6c [6, 9, 12] represent fungal biomass^81,83,84^. The PLFAs were subjected to mild alkaline methanolysis, and the obtained fatty acid methyl esters were separated using a gas chromatograph (Agilent 7820 A) using a capillary column HP-Ultra 2 (cross-linked 5% phenyl-methyl silicone; 25 m, 0.20 mm ID; film thickness 0.33 μm) with hydrogen as the carrier gas. PLFAs were detected using a flame ionization detector (FID) and identified using MIDI Microbial Identification System software (Sherlock 6.1, TSBA 6.1 library; MIDI Inc., Newark, DE, USA). Analysis of variance (ANOVA) followed by Tukey’s honestly significant difference test (Tukey HSD-test test) was performed using STATISTICA 13.3 PL software (StatSoft, Tulsa, USA) to estimate any significant differences between samples taken at different time points. Differences between treatments were considered statistically significant at *p* < 0.05. Data are presented as means ± standard deviation (SD) of three biological replicates.

### Hormonomics analysis

Multiple Phytohormone Profiling by Targeted Metabolomics was applied to measure the phytohormone concentration in barley roots and leaves at 25 DAP (control and drought-treated). Three technical replicates were used for each tissue set. A paired Student’s t-test was used to check for statistically significant differences between samples. ​Phytohormone quantification was conducted following the method described previously^85^. Briefly, approximately 20 mg of fresh plant tissue was homogenized with ice-cold 50% (v/v) acetonitrile, including stable isotope-labeled internal standards. The homogenate was sonicated, extracted at 4 °C, and centrifuged. The supernatant was purified using Oasis HLB solid-phase extraction cartridges, and the eluate was evaporated under nitrogen. The residue was reconstituted in 30% (v/v) acetonitrile and analyzed using ultra-high-performance liquid chromatography coupled with tandem mass spectrometry (UHPLC-MS/MS). Quantification was achieved using the standard isotope dilution method, ensuring accurate measurement of multiple phytohormone classes.

## Electronic supplementary material

Below is the link to the electronic supplementary material.

Supplementary Material 1

Supplementary Material 2

Supplementary Material 3

Supplementary Material 4

Supplementary Material 5

Supplementary Material 6

Supplementary Material 7

Supplementary Material 8

Supplementary Material 9

## Data Availability

All raw data used in this study can be found in the following repositories. Transcriptomic data was deposed into in the Array Express repository (https://www. ebi. ac. uk/) under accession number: E-MTAB-14320Metatranscriptomic data: PRJNA1037705 https://www.ncbi.nlm.nih.gov/sra/PRJNA1037705.

## References

[1] Sato, H., Mizoi, J. & Shinozaki, K. Yamaguchi-Shinozaki, K. Complex plant responses to drought and heat stress under climate change. *Plant J.* **117**, 1873–1892 (2024). 38168757 10.1111/tpj.16612

[2] Xie, W. et al. Decreases in global beer supply due to extreme drought and heat. *Nat. Plants*. **4**, 964–973 (2018). 30323183 10.1038/s41477-018-0263-1

[3] Benitez-Alfonso, Y. et al. Enhancing climate change resilience in agricultural crops. *Curr. Biol.* **33**, R1246–R1261 (2023). 38052178 10.1016/j.cub.2023.10.028

[4] Hirt, H. et al. PlantACT! – how to tackle the climate crisis. *Trends Plant Sci.* **28**, 537–543 (2023). 36740490 10.1016/j.tplants.2023.01.005

[5] Okamoto, M. et al. Activation of dimeric ABA receptors elicits guard cell closure, ABA-regulated gene expression, and drought tolerance. *Proc. Natl. Acad. Sci. U S A*. **110**, 12132–12137 (2013). 23818638 10.1073/pnas.1305919110 PMC3718107

[6] Sah, S. K., Reddy, K. R. & Li, J. Abscisic acid and abiotic stress tolerance in crop plants. *Front. Plant. Sci.* **7**, (2016). 10.3389/fpls.2016.00571 PMC4855980 27200044

[7] Iqbal, S. et al. Phytohormones trigger drought tolerance in crop plants: outlook and future perspectives. *Front. Plant Sci.* **12**, (2022). 10.3389/fpls.2021.799318 PMC8792739 35095971

[8] Jogawat, A. et al. Crosstalk between phytohormones and secondary metabolites in the drought stress tolerance of crop plants: A review. *Physiol. Plant.* **172**, 1106–1132 (2021). 33421146 10.1111/ppl.13328

[9] Calixto, C. P. G. et al. Cold-Dependent expression and alternative splicing of Arabidopsis long Non-coding RNAs. *Front. Plant. Sci.* **10**, 235 (2019). 30891054 10.3389/fpls.2019.00235 PMC6413719

[10] Laloum, T., Martín, G. & Duque, P. Alternative splicing control of abiotic stress responses. *Trends Plant Sci.* **23**, 140–150 (2018). 29074233 10.1016/j.tplants.2017.09.019

[11] Sybilska, E. The cap-binding complex modulates ABA-responsive transcript splicing during germination in barley (Hordeum vulgare). *Scientific Reports* (2024). 10.1038/s41598-024-69373-9 PMC11303550 39107424

[12] Chaudhary, S., Jabre, I., Reddy, A. S. N., Staiger, D. & Syed, N. H. Perspective on alternative splicing and proteome complexity in plants. *Trends Plant Sci.* **24**, 496–506 (2019). 30852095 10.1016/j.tplants.2019.02.006

[13] Lee, H. et al. Genome-wide analysis of alternative splicing events during response to drought stress in tomato (Solanum lycopersicum L). *J. Hortic. Sci. Biotechnol.* **95**, 1–8 (2019).

[14] Song, L. et al. Analysis of whole transcriptome RNA-seq data reveals many alternative splicing events in soybean roots under drought stress conditions. *Genes (Basel)*. **11**, 1520 (2020). 33352659 10.3390/genes11121520 PMC7765832

[15] Filichkin, S., Priest, H. D., Megraw, M. & Mockler, T. C. Alternative splicing in plants: directing traffic at the crossroads of adaptation and environmental stress. *Curr. Opin. Plant. Biol.* **24**, 125–135 (2015). 25835141 10.1016/j.pbi.2015.02.008

[16] Xu, D. et al. Response of the organellar and nuclear (post)transcriptomes of Arabidopsis to drought. *Front. Plant Sci.* **14**, (2023). 10.3389/fpls.2023.1220928 PMC10387551 37528975

[17] Coulter, M. et al. BaRTv2: a highly resolved barley reference transcriptome for accurate transcript-specific RNA ‐seq quantification. *Plant J.* **111**, 1183–1202 (2022). 35704392 10.1111/tpj.15871 PMC9546494

[18] de Vries, F. T., Griffiths, R. I., Knight, C. G., Nicolitch, O. & Williams, A. Harnessing rhizosphere microbiomes for drought-resilient crop production. *Science* **368**, 270–274 (2020). 32299947 10.1126/science.aaz5192

[19] Finkel, O. M., Castrillo, G., Herrera Paredes, S., Salas González, I. & Dangl, J. L. Understanding and exploiting plant beneficial microbes. *Curr. Opin. Plant. Biol.* **38**, 155–163 (2017). 28622659 10.1016/j.pbi.2017.04.018 PMC5561662

[20] Dodd, I., Zinovkina, N., Safronova, V. & Belimov, A. a. Rhizobacterial mediation of plant hormone status. *Ann. Appl. Biol.* **157**, 361–379 (2010).

[21] Jochum, M. D. et al. Bioprospecting plant Growth-Promoting rhizobacteria that mitigate drought stress in grasses. *Front. Microbiol.* **10**, (2019). 10.3389/fmicb.2019.02106 PMC6747002 31552009

[22] Naveed, M., Hussain, M. B., Zahir, Z. A., Mitter, B. & Sessitsch, A. Drought stress amelioration in wheat through inoculation with Burkholderia phytofirmans strain PsJN. *Plant. Growth Regul.* **2**, 121–131 (2014).

[23] Bento, V. A. et al. The impact of climate change in wheat and barley yields in the Iberian Peninsula. *Sci. Rep.* **11**, 15484 (2021). 34326411 10.1038/s41598-021-95014-6 PMC8322258

[24] Bertholdsson, N. O. Screening for barley waterlogging tolerance in nordic barley cultivars (Hordeum vulgare L.) using chlorophyll fluorescence on Hydroponically-Grown plants. *Agronomy* **3**, 376–390 (2013).

[25] Dawson, I. K. et al. Barley: a translational model for adaptation to climate change. *New. Phytol*. **206**, 913–931 (2015). 25605349 10.1111/nph.13266

[26] Zhang, Y. et al. Abscisic acid mediates barley rhizosheath formation under mild soil drying by promoting root hair growth and auxin response. *Plant. Cell. Environ.* **44**, 1935–1945 (2021). 33629760 10.1111/pce.14036

[27] Tartaglia, M. et al. Metatranscriptomics of pastures under drought stress show a rhizospheric meta-organism. *Rhizosphere* **26**, 100687 (2023).

[28] Xu, L. et al. Genome-resolved metagenomics reveals role of iron metabolism in drought-induced rhizosphere Microbiome dynamics. *Nat. Commun.* **12**, 3209 (2021). 34050180 10.1038/s41467-021-23553-7 PMC8163885

[29] Alegria Terrazas, R. et al. A footprint of plant eco-geographic adaptation on the composition of the barley rhizosphere bacterial microbiota. *Sci. Rep.* **10**, 12916 (2020). 32737353 10.1038/s41598-020-69672-x PMC7395104

[30] Escudero-Martinez, C. et al. Identifying plant genes shaping microbiota composition in the barley rhizosphere. *Nat. Commun.* **13**, 3443 (2022). 35710760 10.1038/s41467-022-31022-y PMC9203816

[31] Gaillochet, C. et al. A molecular network for functional versatility of HECATE transcription factors. *Plant J.* **95**, 57–70 (2018). 29667268 10.1111/tpj.13930

[32] Gremski, K., Ditta, G. & Yanofsky, M. F. The HECATE genes regulate female reproductive tract development in Arabidopsis thaliana. *Development* **134**, 3593–3601 (2007). 17855426 10.1242/dev.011510

[33] Dubois, M. et al. The ETHYLENE RESPONSE factors ERF6 and ERF11 antagonistically regulate Mannitol-Induced growth Inhibition in Arabidopsis. *Plant Physiol.* **169**, 166–179 (2015). 25995327 10.1104/pp.15.00335 PMC4577380

[34] Ahmad, R. et al. GOLDEN2-LIKE transcription factors regulate *WRKY40* expression in response to abscisic acid. *Plant. Physiol.* **179**, 1844–1860 (2019). 30723180 10.1104/pp.18.01466 PMC6446771

[35] Chen, H. et al. Roles of arabidopsis WRKY18, WRKY40 and WRKY60 transcription factors in plant responses to abscisic acid and abiotic stress. *BMC Plant Biol.* **10**, 281 (2010). 21167067 10.1186/1471-2229-10-281 PMC3023790

[36] Lotkowska, M. E. et al. The Arabidopsis transcription factor MYB112 promotes anthocyanin formation during salinity and under high light stress. *Plant Physiol.* **169**, 1862–1880 (2015). 26378103 10.1104/pp.15.00605 PMC4634054

[37] Nawy, T. et al. Transcriptional profile of the Arabidopsis root quiescent center. *Plant. Cell.* **17**, 1908–1925 (2005). 15937229 10.1105/tpc.105.031724 PMC1167541

[38] Milne, L. et al. EORNA, a barley gene and transcript abundance database. *Sci. Data*. **8**, 90 (2021). 33767193 10.1038/s41597-021-00872-4 PMC7994555

[39] Pande, P. M., Azarbad, H., Tremblay, J., St-Arnaud, M. & Yergeau, E. Metatranscriptomic response of the wheat holobiont to decreasing soil water content. *ISME COMMUN.* **3**, 1–13 (2023). 37061589 10.1038/s43705-023-00235-7 PMC10105728

[40] Rosado-Porto, D. et al. Soil metatranscriptome demonstrates a shift in C, N, and S metabolisms of a grassland ecosystem in response to elevated atmospheric CO2. *Front. Microbiol.* **13**, (2022). 10.3389/fmicb.2022.937021 PMC9445814 36081791

[41] Sharma, P. K. et al. Comparative metatranscriptome analysis revealed broad response of microbial communities in two soil types, agriculture versus organic soil. *J. Genet. Eng. Biotechnol.* **17**, 6 (2019). 31659568 10.1186/s43141-019-0006-3 PMC6821142

[42] Feng, Y., Zhao, Y., Li, Y., Zhou, J. & Shi, H. Improving photosynthesis and drought tolerance in Nicotiana tabacum L. by foliar application of Salicylic acid. *All Life*. **16**, 2224936 (2023).

[43] Wang, Y. et al. Transcriptomic and physiological responses of contrasting maize genotypes to drought stress. *Front. Plant Sci.* **13**, (2022). 10.3389/fpls.2022.928897 PMC9381927 35991451

[44] Karlova, R., Boer, D., Hayes, S. & Testerink, C. Root plasticity under abiotic stress. *Plant Physiol.* **187**, 1057–1070 (2021). 34734279 10.1093/plphys/kiab392 PMC8566202

[45] Chimungu, J. G., Brown, K. M. & Lynch, J. P. Large root cortical cell size improves drought tolerance in Maize1[C][W][OPEN]. *Plant. Physiol.* **166**, 2166–2178 (2014). 25293960 10.1104/pp.114.250449 PMC4256844

[46] Hazman, M. & Brown, K. M. Progressive drought alters architectural and anatomical traits of rice roots. *Rice* **11**, 62 (2018). 30511228 10.1186/s12284-018-0252-z PMC6277260

[47] Beyer, S. et al. Loci and candidate genes controlling root traits in wheat seedlings—a wheat root GWAS. *Funct. Integr. Genomics*. **19**, 91–107 (2019). 30151724 10.1007/s10142-018-0630-z

[48] Ling, Q. & Jarvis, P. Regulation of Chloroplast protein import by the ubiquitin E3 ligase SP1 is important for stress tolerance in plants. *Curr. Biol.* **25**, 2527–2534 (2015). 26387714 10.1016/j.cub.2015.08.015 PMC4598742

[49] Pratelli, R. et al. The ubiquitin E3 ligase LOSS OF GDU2 is required for GLUTAMINE DUMPER1-Induced amino acid secretion in Arabidopsis. *Plant Physiol.* **158**, 1628–1642 (2012). 22291198 10.1104/pp.111.191965 PMC3320174

[50] Niu, X. et al. Identification of wheat DREB genes and functional characterization of TaDREB3 in response to abiotic stresses. *Gene* **740**, 144514 (2020). 32112985 10.1016/j.gene.2020.144514

[51] Peixoto-Junior, R. F. et al. Overexpression of ScMYBAS1 alternative splicing transcripts differentially impacts biomass accumulation and drought tolerance in rice Transgenic plants. *PLOS ONE*. **13**, e0207534 (2018). 30517137 10.1371/journal.pone.0207534 PMC6281192

[52] Ganie, S. A. & Reddy, A. S. N. Stress-Induced changes in alternative splicing landscape in rice: functional significance of splice isoforms in stress tolerance. *Biology (Basel)*. **10**, 309 (2021). 33917813 10.3390/biology10040309 PMC8068108

[53] Harb, A. et al. The effect of drought on transcriptome and hormonal profiles in barley genotypes with contrasting drought tolerance. *Front. Plant. Sci.* **11**, 618491 (2020). 33424910 10.3389/fpls.2020.618491 PMC7786106

[54] Kishor, P. B. K., Tiozon, R. N., Fernie, A. R. & Sreenivasulu, N. Abscisic acid and its role in the modulation of plant growth, development, and yield stability. *Trends Plant Sci.* **27**, 1283–1295 (2022). 36100537 10.1016/j.tplants.2022.08.013

[55] Müller, M. & Munné-Bosch, S. Hormonal impact on photosynthesis and photoprotection in plants. *Plant. Physiol.* **185**, 1500–1522 (2021). 33793915 10.1093/plphys/kiaa119 PMC8133604

[56] Collin, A., Daszkowska-Golec, A., Kurowska, M. & Szarejko, I. Barley ABI5 (Abscisic acid INSENSITIVE 5) is involved in abscisic acid-Dependent drought response. *Front. Plant. Sci.* **11**, 1138 (2020). 32849699 10.3389/fpls.2020.01138 PMC7405899

[57] Yoshida, T. et al. AREB1, AREB2, and ABF3 are master transcription factors that cooperatively regulate ABRE-dependent ABA signaling involved in drought stress tolerance and require ABA for full activation. *Plant J.* **61**, 672–685 (2010). 19947981 10.1111/j.1365-313X.2009.04092.x

[58] Marzec, M. et al. Barley Strigolactone signalling mutant *hvd14.d* reveals the role of Strigolactones in abscisic acid-dependent response to drought. *Plant. Cell. Environ.* **43**, 2239–2253 (2020). 32501539 10.1111/pce.13815

[59] Seiler, C. et al. Abscisic acid flux alterations result in differential abscisic acid signaling responses and impact assimilation efficiency in barley under terminal drought stress. *Plant. Physiol.* **164**, 1677–1696 (2014). 24610749 10.1104/pp.113.229062 PMC3982733

[60] Ogawa, D. et al. Acetic-acid-induced jasmonate signaling in root enhances drought avoidance in rice. *Sci. Rep.* **11**, 6280 (2021). 33737547 10.1038/s41598-021-85355-7 PMC7973560

[61] Ilyas, M. et al. Drought tolerance strategies in plants: A mechanistic approach. *J. Plant Growth Regul.* **40**, (2021).

[62] Kim, J. Y. et al. Distinct identities of leaf phloem cells revealed by single cell transcriptomics. *Plant. Cell.* **33**, 511–530 (2021). 33955487 10.1093/plcell/koaa060 PMC8136902

[63] Wang, X. et al. Abscisic acid and jasmonic acid are involved in drought priming-induced tolerance to drought in wheat. *Crop J.* **9**, 120–132 (2021).

[64] Liu, Y. Wirén, N. Integration of nutrient and water availabilities via auxin into the root developmental program. *Curr. Opin. Plant. Biol.* **65**, 102117 (2022). von. 34624806 10.1016/j.pbi.2021.102117

[65] Kalve, S. et al. Osmotic stress inhibits leaf growth of Arabidopsis thaliana by enhancing ARF-mediated auxin responses. *New. Phytol*. **226**, 1766–1780 (2020). 32077108 10.1111/nph.16490

[66] Janiak, A. et al. No time to waste: transcriptome study reveals that drought tolerance in barley May be attributed to stressed-Like expression patterns that exist before the occurrence of stress. *Front. Plant. Sci.* **8**, 2212 (2018). 29375595 10.3389/fpls.2017.02212 PMC5767312

[67] Mahalingam, R. et al. Heat and drought induced transcriptomic changes in barley varieties with contrasting stress response phenotypes. *Front. Plant. Sci.* **13**, (2022). 10.3389/fpls.2022.1066421 PMC9772561 36570886

[68] Alawiye, T. & Babalola, O. Bacterial diversity and community structure in typical plant rhizosphere. *Diversity* **11**, 179 (2019).

[69] Filippidou, S. et al. A combination of extreme environmental conditions favor the prevalence of Endospore-Forming Firmicutes. *Front. Microbiol.* **7**, 1707 (2016). 27857706 10.3389/fmicb.2016.01707 PMC5094177

[70] Martínez-Viveros, O., Jorquera, M. A., Crowley, D. E., Gajardo, G. & Mora, M. L. MECHANISMS AND PRACTICAL CONSIDERATIONS INVOLVED IN PLANT GROWTH PROMOTION BY RHIZOBACTERIA. *J. Soil. Sci. Plant. Nutr.* **10**, 293–319 (2010).

[71] Rousk, J., Brookes, P. & Bååth, E. The microbial PLFA composition as affected by pH in an arable soil. *Soil Biol. Biochem.* **42**, 516–520 (2010).

[72] Santos-Medellín, C., Edwards, J., Liechty, Z., Nguyen, B. & Sundaresan, V. Drought stress results in a Compartment-Specific restructuring of the rice Root-Associated microbiomes. *mBio* **8**, e00764–e00717 (2017). 28720730 10.1128/mBio.00764-17 PMC5516253

[73] Patro, R., Duggal, G., Love, M. I., Irizarry, R. A. & Kingsford, C. Salmon provides fast and bias-aware quantification of transcript expression. *Nat. Methods*. **14**, 417–419 (2017). 28263959 10.1038/nmeth.4197 PMC5600148

[74] Sebestyén, E., Zawisza, M. & Eyras, E. Detection of recurrent alternative splicing switches in tumor samples reveals novel signatures of cancer. *Nucleic Acids Res.* **43**, 1345–1356 (2015). 25578962 10.1093/nar/gku1392 PMC4330360

[75] Tian, F., Yang, D. C., Meng, Y. Q., Jin, J. & Gao, G. PlantRegMap: charting functional regulatory maps in plants. *Nucleic Acids Res.* **gkz1020**10.1093/nar/gkz1020 (2019). 10.1093/nar/gkz1020 PMC7145545 31701126

[76] Kieser, S., Brown, J., Zdobnov, E. M., Trajkovski, M. & McCue, L. A. ATLAS: a snakemake workflow for assembly, annotation, and genomic Binning of metagenome sequence data. *BMC Bioinform.* **21**, 257 (2020). 10.1186/s12859-020-03585-4 PMC7310028 32571209

[77] Nurk, S., Meleshko, D., Korobeynikov, A. & Pevzner, P. A. MetaSPAdes: a new versatile metagenomic assembler. *Genome Res.* **27**, 824–834 (2017). 28298430 10.1101/gr.213959.116 PMC5411777

[78] Huerta-Cepas, J. et al. EggNOG 5.0: a hierarchical, functionally and phylogenetically annotated orthology resource based on 5090 organisms and 2502 viruses. *Nucleic Acids Res.* **47**, D309–D314 (2019). 30418610 10.1093/nar/gky1085 PMC6324079

[79] Mallick, H. et al. Multivariable association discovery in population-scale meta-omics studies. *PLoS Comput. Biol.* **17**, e1009442 (2021). 34784344 10.1371/journal.pcbi.1009442 PMC8714082

[80] Wu, T. et al. ClusterProfiler 4.0: A universal enrichment tool for interpreting omics data. *Innov.* **2**, 100141 (2021). 10.1016/j.xinn.2021.100141 PMC8454663 34557778

[81] Pennanen, T. et al. Structure of the microbial communities in coniferous forest soils in relation to site fertility and stand development stage. *Microb. Ecol.* **38**, 168–179 (1999). 10441709 10.1007/s002489900161

[82] Płociniczak, T., Sinkkonen, A., Romantschuk, M. & Piotrowska-Seget, Z. Characterization of Enterobacter intermedius MH8b and its use for the enhancement of heavy metals uptake by Sinapis alba L. *Appl. Soil. Ecol.* **63**, 1–7 (2013).

[83] Bååth, E. & Anderson, T. H. Comparison of soil fungal/bacterial ratios in a pH gradient using physiological and PLFA-based techniques. *Soil Biol. Biochem.* **35**, 955–963 (2003).

[84] Frostegard, A. & Bååth, E. The use of phospholipid fatty acid analysis to estimate bacterial and fungal biomass in soil. *Biol. Fertil. Soils*. **22**, 59–65 (1996).

[85] Šimura, J. et al. Plant hormonomics: multiple phytohormone profiling by targeted metabolomics. *Plant. Physiol.* **177**, 476–489 (2018). 29703867 10.1104/pp.18.00293 PMC6001343

[86] Schneider, M. et al. Transcriptome profiling of barley and tomato shoot and root meristems unravels physiological variations underlying photoperiodic sensitivity. *PLoS One*. **17**, e0265981 (2022). 36095002 10.1371/journal.pone.0265981 PMC9467324

