# Supplementary material for: Hormonal and transcriptomic regulation of drought adaptation in barley roots and leaves
Source: Sci Rep. 2025 May 11;15:16368. doi: 10.1038/s41598-025-01590-2 (PMC12066718; doi:10.1038/s41598-025-01590-2)
Supplement: Supplementary file 4 — Supplementary Material 4 [file 41598_2025_1590_MOESM4_ESM.docx]

**Supplementary Material S8**: Macro- and micro-element composition of soil used in drought experiment.

| **Compound** | **Amount** | **Unit** |  |
| --- | --- | --- | --- |
| pH in KCl | 5,2 ± 0,3 |  |  |
|  |  |  |  |
|  |  |  |  |
| Absorbable phosphorus as P_2_O_5_ | 16,6 ± 3,7 | mg/100g |  |
|  |  |  |  |
|  |  |  |  |
| Absorbable magnesium | 4,8 ± 0,5 | mg/100g |  |
|  |  |  |  |
|  |  |  |  |
| Absorbable potassium as K_2_O | 6,8 ± 0,8 | mg/100g |  |
|  |  |  |  |
|  |  |  |  |
| Nitrogen | below 0,1 | % |  |
|  |  |  |  |
|  |  |  |  |
| Organic coal | 0,78 ± 0,16 | % |  |
|  |  |  |  |
|  |  |  |  |
| Sulphur | below 0,1 | % |  |
|  |  |  |  |
|  |  |  |  |
| Humus | 1,34 ± 0,27 | % |  |
|  |  |  |  |
|  |  |  |  |
| Boron | 0,3 ± 0,1 | mg/kg |  |
|  |  |  |  |
|  |  |  |  |
| Copper | 2,0 ± 0,3 | mg/kg |  |
|  |  |  |  |
|  |  |  |  |
| Iron | 526 ±68 | mg/kg |  |
|  |  |  |  |
|  |  |  |  |
| Manganese | 95 ± 12 | mg/kg |  |
|  |  |  |  |
|  |  |  |  |
| Molybdenum | below 0,06 | mg/kg |  |
|  |  |  |  |
|  |  |  |  |
| Zinc | 5,7 ± 0,9 | mg/kg |  |
|  |  |  |  |
|  |  |  |  |

Composition of nutrient solution used for soil supplementation used in drought experiment. Each box was supplemented with 90 ml of mineral compound mixture and 10 ml of additional nitrate solution.

| **Mixture of mineral compounds** | |
| --- | --- |
| **Compound** | **Weight per 1 L (g)** |
| NH_4_NO_3_ | 34.3 |
| KH_2_PO_4_ | 40.8 |
| K_2_SO_4_ | 10 |
| MgSO_4_×7H_2_O | 61.5 |
| H_3_BO_3_ | 0.05 |
| CuSO_4_ | 0.02 |
| MnSO_4_×H_2_O | 0.01 |
| Fe(C_6_H_5_O_7_)×3H_2_O | 0.5 |

**Additional nitrate solution**

3.43 g of NH_4_NO_3_ was dissolved in 10 ml of water.
