# Supplementary material for: Hormonal and transcriptomic regulation of drought adaptation in barley roots and leaves
Source: Sci Rep. 2025 May 11;15:16368. doi: 10.1038/s41598-025-01590-2 (PMC12066718; doi:10.1038/s41598-025-01590-2)
Supplement: Supplementary file 9 — Supplementary Material 9 [file 41598_2025_1590_MOESM9_ESM.docx]

**Supplementary Figure X.** The phenotype of cv. ‘Sebastian’ seedlings on 10 DAP and on 15 DAP and 25 DAP under control conditions and drought.


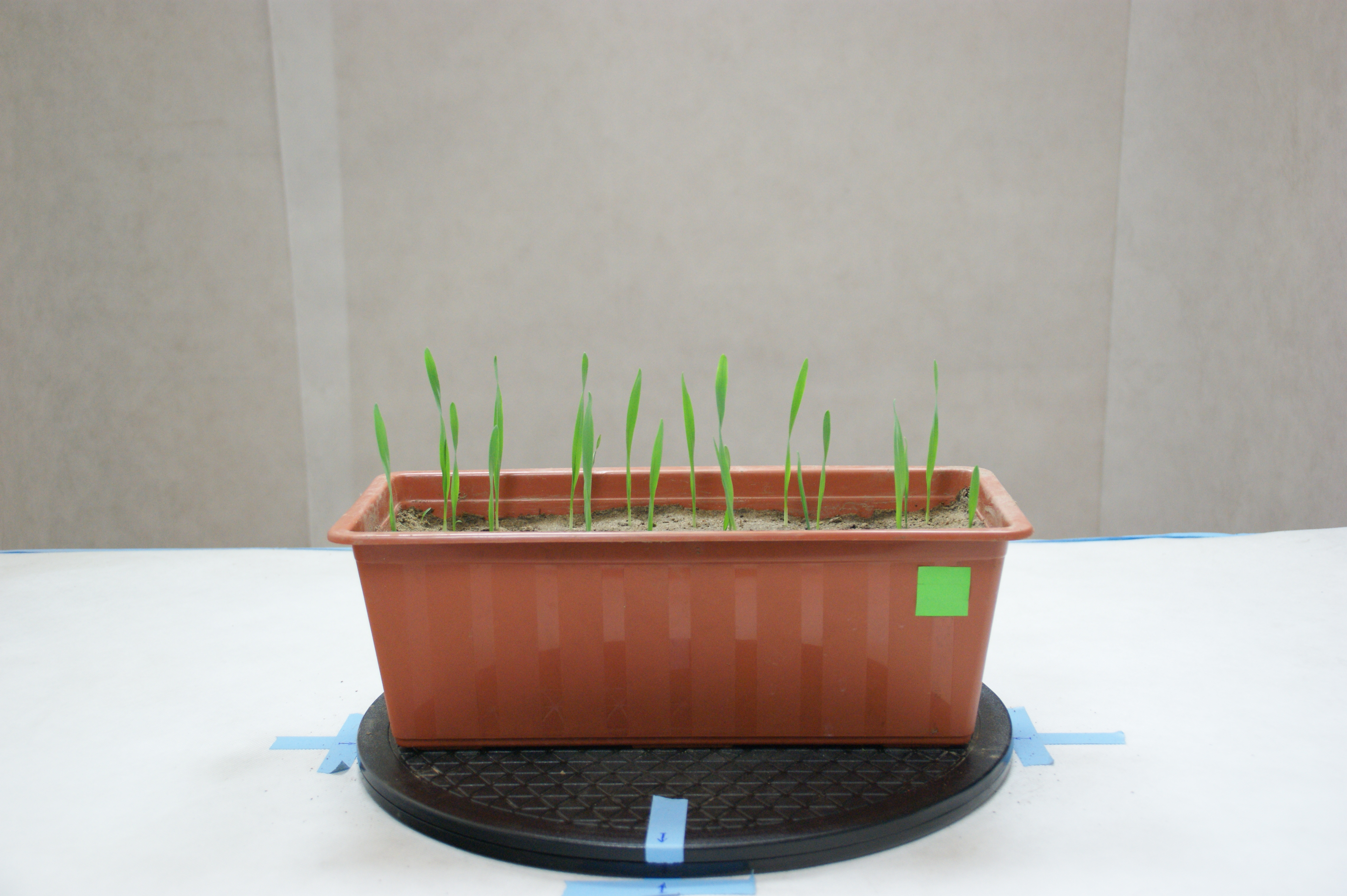

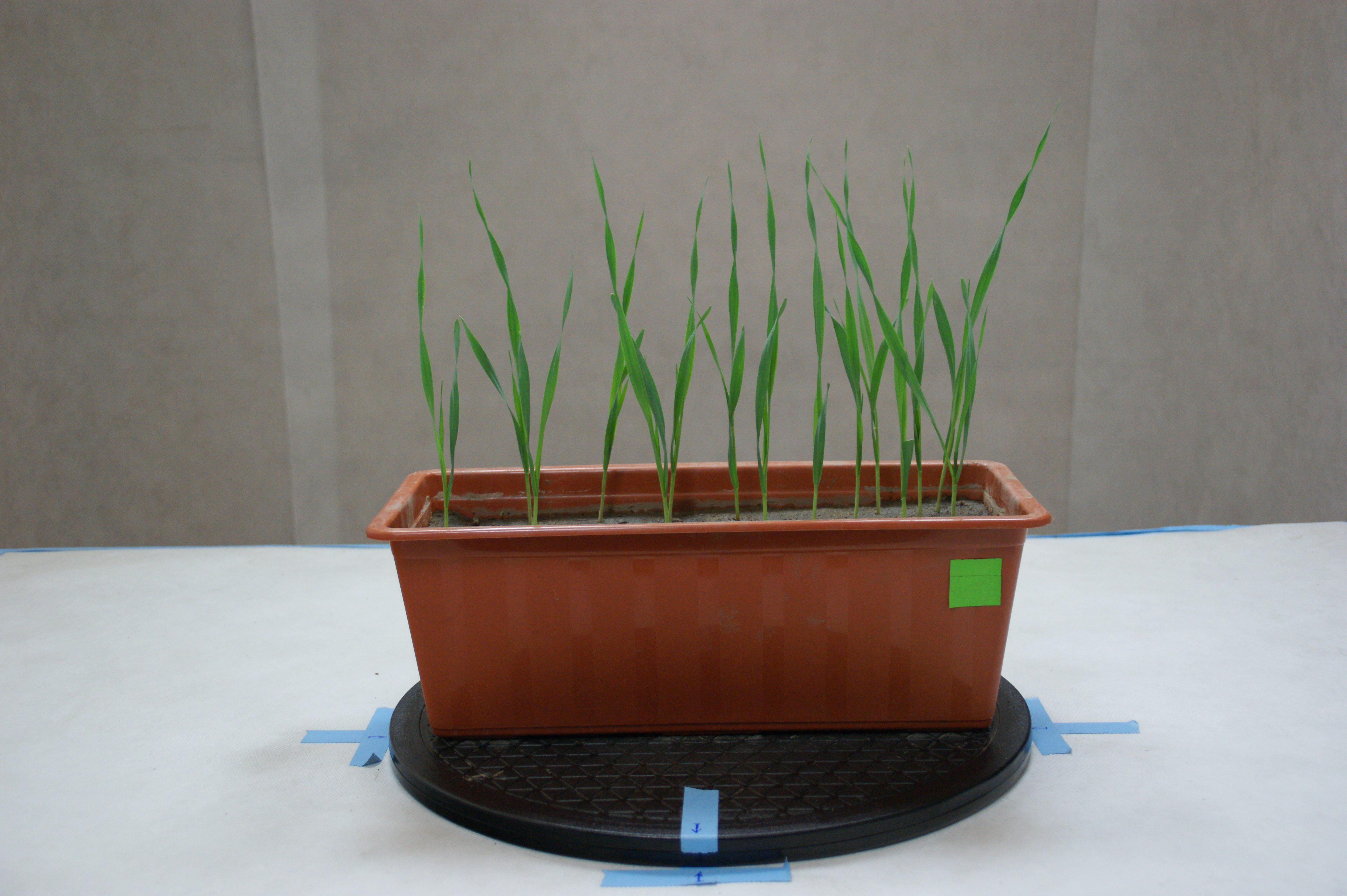

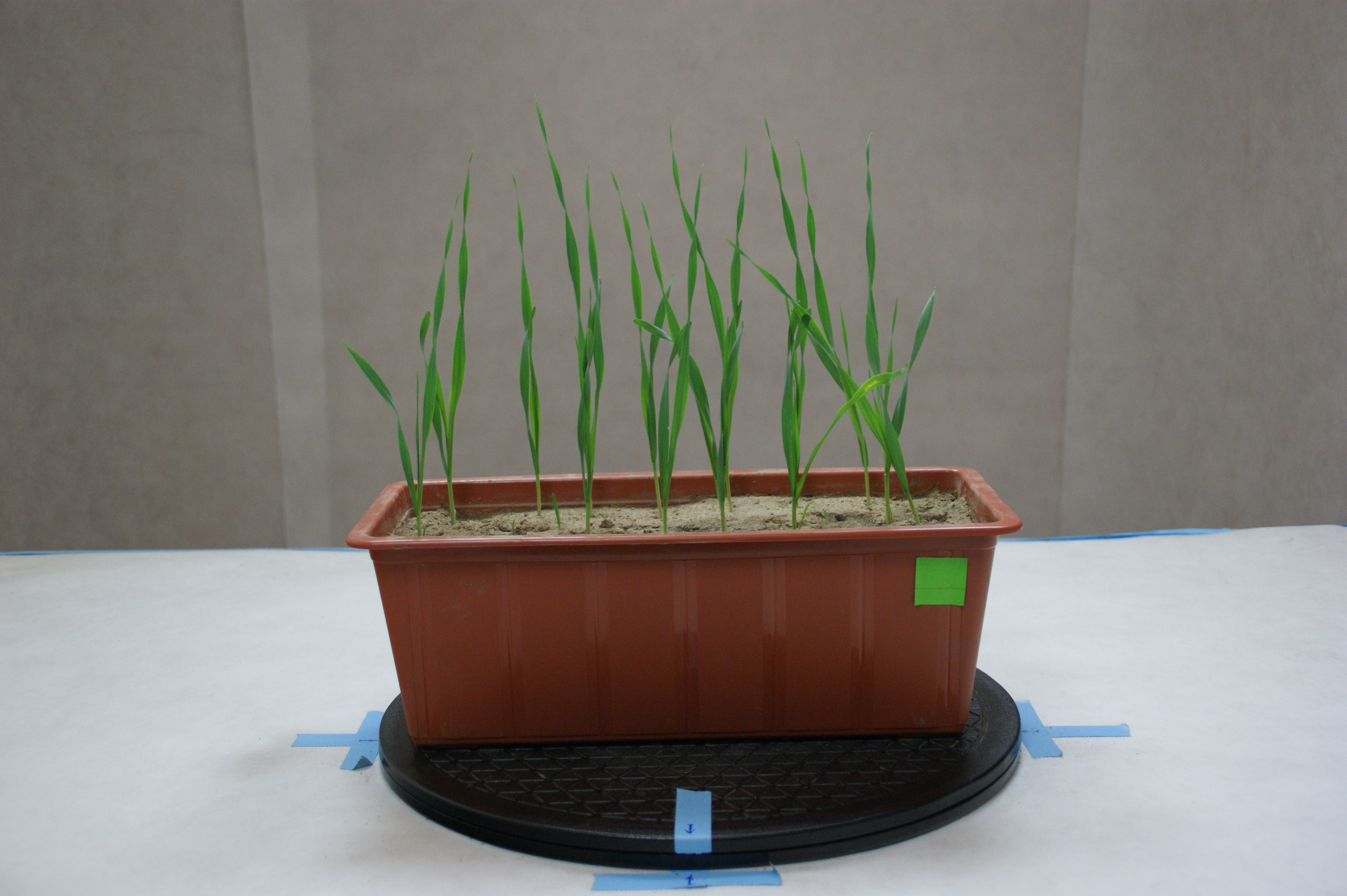

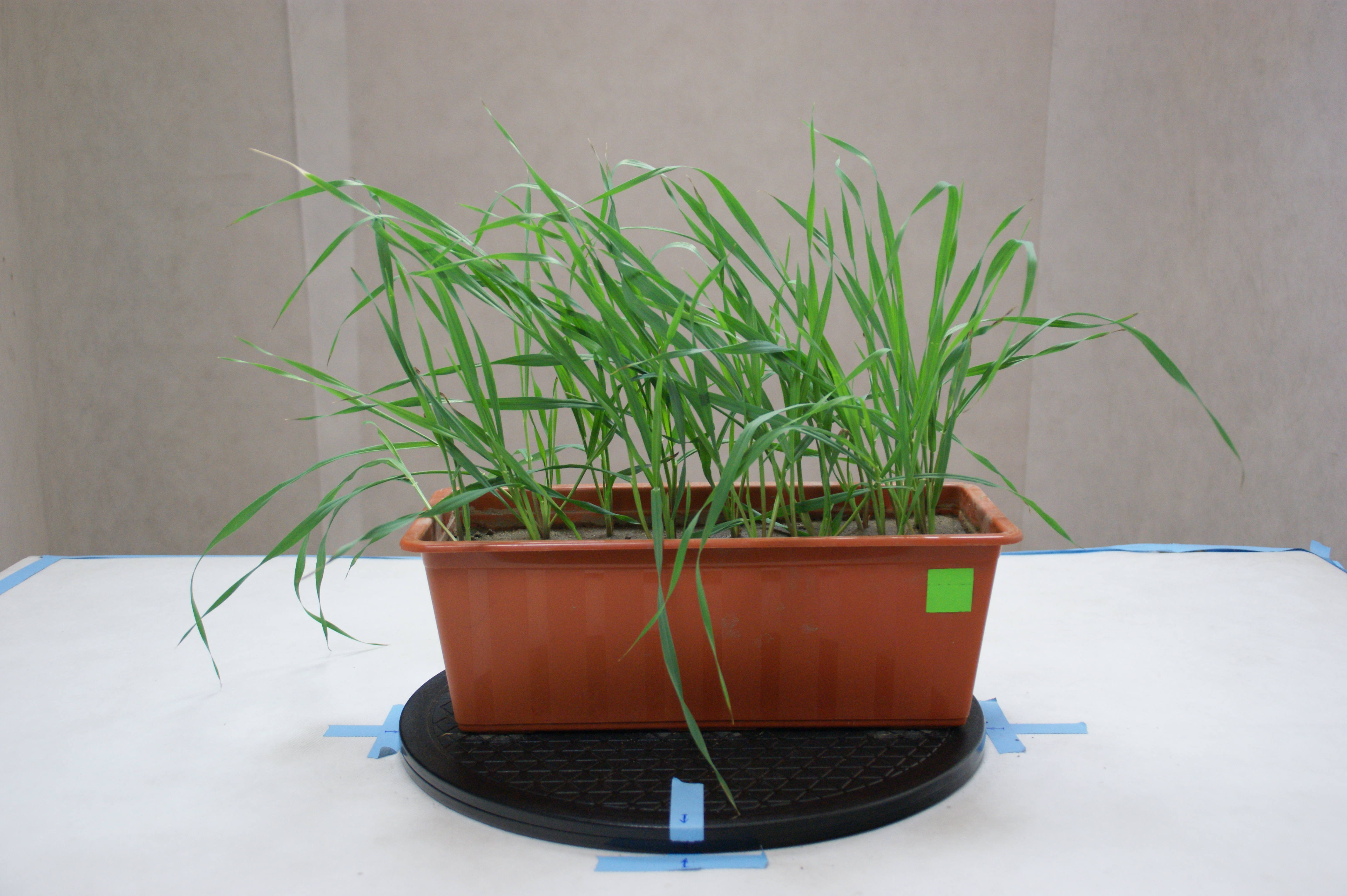

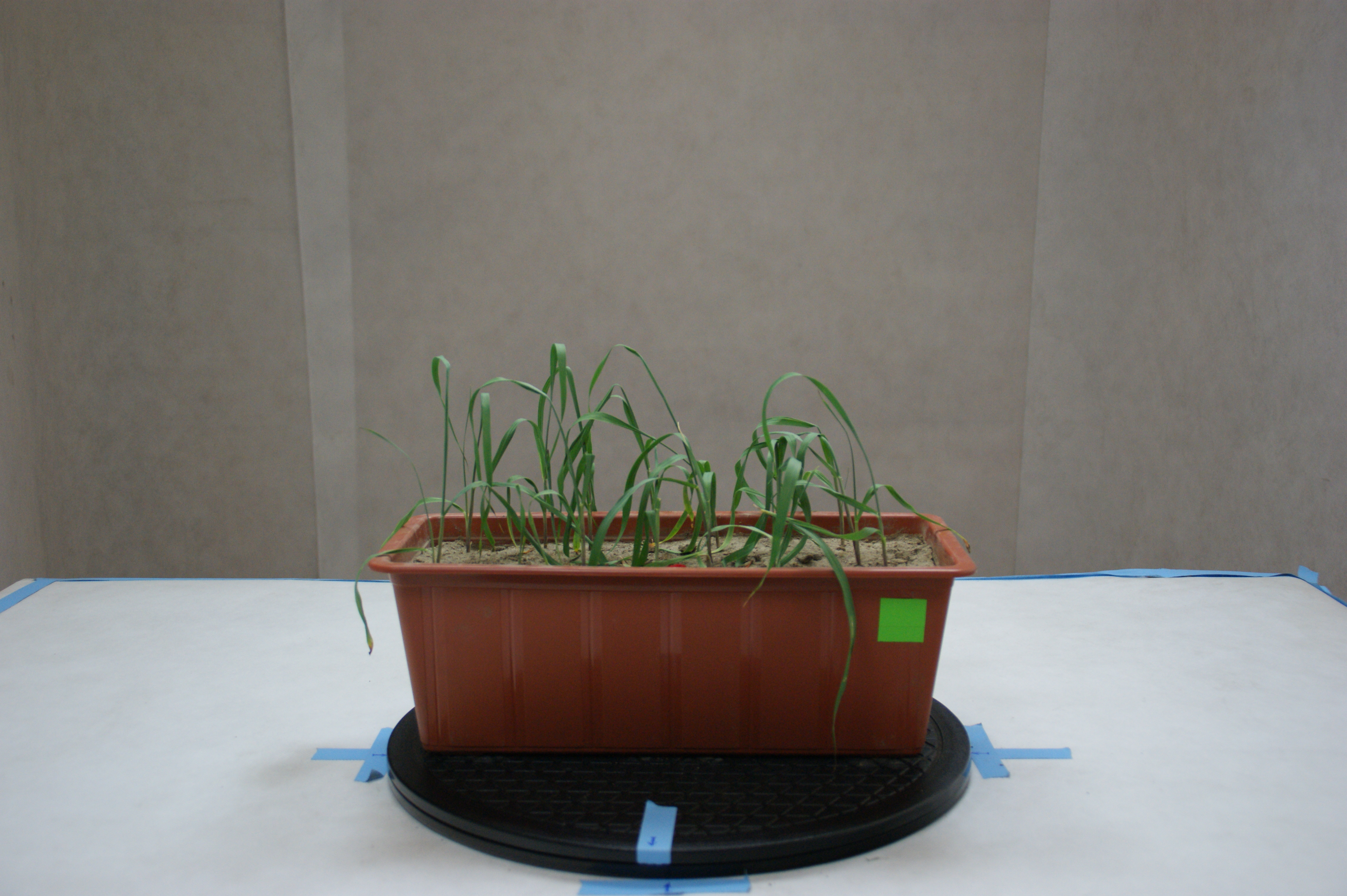


**10 DAP**

**Control**

**Drought**

**15 DAP**

**25 DAP**
